# Wavelet Transform‐Based Atomic Force Microscopy: A Computational Paradigm for Dynamic Nanoscale Imaging and Characterisation

**DOI:** 10.1002/smsc.70350

**Published:** 2026-07-26

**Authors:** Pardis Biglarbeigi, Navneet Soin, Amit Kumar, Dewar Finlay, Amir Farokh Payam

**Affiliations:** ^1^ Department of Pharmacology & Therapeutics University of Liverpool Liverpool England UK; ^2^ PulseAI Ltd Belfast UK; ^3^ School of Science Computing and Emerging Technologies (SoSCET) Swinburne University of Technology Hawthorn Victoria Australia; ^4^ School of Science RMIT University Melbourne Victoria Australia; ^5^ School of Mathematics and Physics Centre for Quantum Materials and Technologies (CQMT) Queen's University Belfast Belfast UK; ^6^ School of Engineering Ulster University Belfast Northern Ireland UK

**Keywords:** Atomic Force Microscopy, image fusion, multi‐frequency dynamics, nonlinear harmonics, temporal resolution, time‐resolved Kelvin Probe Force Microscopy, wavelet, wavelet transform

## Abstract

Wavelet transform‐based atomic force microscopy (WT‐AFM) marks a significant paradigm shift in nanoscale imaging by enabling real‐time, simultaneous computational analysis of tip–sample interactions in both the time and frequency domains. Unlike conventional AFM approaches that are limited to steady‐state, single‐frequency responses—the WT‐AFM directly applies wavelet transform techniques to the raw cantilever deflection signal, providing direct access to non‐linear, transient and multi‐frequency dynamics that remain obscured in traditional modalities. The WT‐AFM framework integrates high‐speed data acquisition, wavelet decomposition, adaptive noise filtering and a custom unsupervised image fusion algorithm to generate high‐contrast, information‐rich maps of nanoscale heterogeneity. Coupled with digital twin simulations, this approach bridges experimental measurement with underlying materials physics, offering a powerful interpretative framework for dynamic spectral features. Beyond surpassing the existing multi‐frequency AFM techniques in temporal resolution, bandwidth and sensitivity to non‐steady‐state behaviour, the WT‐AFM establishes a dynamic platform for high‐speed force mapping, time‐resolved Kelvin Probe Force Microscopy and the investigation of complex viscoelastic or multi‐layer systems. This perspective highlights how WT‐AFM stands to redefine nanoscale characterisation by extending AFM as a computational dynamic platform for probing the temporal evolution of electronic processes, transient interactions and functional heterogeneity at the nanoscale.

## Graphical abstract

Wavelet transform‐based AFM enables real‐time nanoscale imaging by applying wavelet analysis to raw cantilever deflection signals. It reveals non‐linear, transient and multi‐frequency dynamics beyond conventional steady‐state methods. Integrated with adaptive filtering and digital twin modelling, it produces high‐resolution, information‐rich maps of nanoscale heterogeneity and functional material behaviour at nanoscale systems.

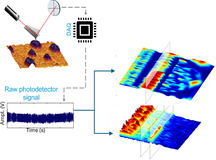

## 1 Introduction

The discovery, understanding and use of advanced materials has become a cornerstone of modern nanotechnology, driving transformative progress in materials science, electronics and photonics [1, 2]. Breakthroughs such as strain‐tuned electronics [3], photo‐capacitive biointerfaces [4, 5], perovskite photovoltaics [6] and human–machine interfaces [7] fundamentally rely on the ability to understand and control matter at the nanoscale. The static and dynamic properties of quantum, plasmonic and van der Waals heterostructures, particularly their surface potential (SP), charge distribution and interfacial mechanics, govern their electronic, mechanical, chemical and optical behaviour [8, 9, 10]. At this scale, surface interactions dictate adsorption, stiffness, reactivity and energy dissipation, shaping phenomena from catalytic efficiency and charge transport to light–matter coupling. These interfacial mechanisms bridge disciplines and blur interdisciplinary boundaries, linking the physics of nanoscale forces with chemical reactivity and biomechanical function. Consequently, unlocking the full potential of emerging material systems demands microscopy techniques capable of capturing nanoscale processes with both high spatial and temporal resolution to reveal transient dynamics and interfacial phenomena beyond the limits of conventional imaging modalities.

Among the vast array of spectroscopy and microscopy tools, atomic force microscopy (AFM) has emerged as the most versatile and powerful method for probing and manipulating materials at the nanoscale [11, 12]. In contrast to electron or optical microscopy, AFM readily operates under ambient or physiological conditions with minimal sample preparation while simultaneously offering sub‐nanometre spatial resolution and access to mechanical, electrical and functional properties [11, 12]. Beyond conventional static topographic imaging, AFM can capture harmonic and multi‐frequency signals arising from non‐linear tip–sample interactions, providing direct measurements of elasticity, adhesion, viscoelasticity and dissipation [13]. The ability to map the three‐dimensional spatial variations of these parameters transforms AFM into a comprehensive platform for correlative nanoscale imaging of physical and functional heterogeneity. Furthermore, the recent advances in machine learning (ML)‐based workflows and wavelet transform (WT)‐based analysis further enhance AFM's capacity to denoise, extract salient features and interpret complex multimodal data with high fidelity [8, 14, 15, 16]. These attributes collectively position AFM as one of the more informative and adaptable nanoscale characterisation tools for advancing materials discovery in electronics, photonics and a range of emerging technologies.

Traditional dynamic AFM modalities, such as amplitude modulation AFM (AM‐AFM or tapping mode) and more advanced multi‐frequency approaches, rely predominantly on lock‐in amplifiers (LIAs) to demodulate the cantilever oscillation signals [13]. These LIAs extract amplitude and phase information primarily at the cantilever's fundamental resonance frequency or, in multi‐frequency implementations, a selected set of eigenmodes corresponding to mode sensitivity [13]. However, this reliance imposes inherent constraints on both temporal resolution (typically 5–10 ms) and frequency bandwidth (Bandwidth (Hz)= 1/(4πτ), where τ is the time constant of the low‐pass filter used after demodulation), effectively averaging out transient dynamics and non‐linear interactions between the AFM tip and the sample surface [17]. In the case of multi‐frequency approaches, attempting to capture a broad spectrum of eigenmodes and higher‐order harmonics requires deploying multiple LIAs in parallel. This significantly increases the instrumental complexity and introduces severe phase‐synchronisation challenges, creating a hardware bottleneck for extracting broadband spatiotemporal information.

A common strategy to overcome these limitations is the deployment of high‐speed LIAs; however, these suffer from increased noise bandwidth, discrete frequency coverage and necessitate the use of low‐pass filters to correct phase mismatches. This filtering inevitably leads to information loss [17], reduced effective bandwidth [18] and suppression of transient responses that evolve faster than the feedback response or the cantilever response time to reach a steady state [19, 20]. To address these shortcomings, several alternative demodulation approaches have been proposed, including synchronous detection (LIA‐based mixing) [21], asynchronous methods (e.g., peak‐hold detection) [22] and adaptive estimators based on Lyapunov and Kalman filtering frameworks [19]. While these approaches improve measurement speed and robustness, they also introduce new challenges: mixing‐based detection may introduce upper sidebands if filtering is insufficient and compromise signal integrity; while Lyapunov and Kalman filters, despite their compensatory capabilities, are algorithmically complex and are sensitive to broadband noise outside the carrier frequency. A comprehensive assessment of these high‐speed demodulation schemes is provided in Ruppert et al. [19].

Despite these innovations, existing demodulation techniques are optimised primarily for linear systems and are best suited for rapid amplitude and phase estimation under stable conditions. Crucially, they lack the ability to capture multi‐dimensional, non‐linear and transient signal features that arise from real‐time cantilever–surface interactions. This includes complex mode coupling, higher‐order harmonics and rapidly changing interfacial forces, all of which are critical to fully understanding nanoscale material behaviour [17, 23].

While WTs have previously been applied in AFM for image analysis [24], studying eigenmodes and energy dissipation in force spectroscopy [14, 25, 26, 27, 28, 29, 30], passive micro‐rheology in living myoblast and the assessment of higher harmonic amplitudes in tapping mode [15, 31], our body of work is the first to introduce a fundamentally new approach to dynamic AFM data acquisition and imaging based on the application of WT directly to the photo‐detector data stream [32, 33, 34, 35]. This approach provides the opportunity for exploration of the transient response of the cantilever, analysis and imaging of the dynamics of amplitude and phase of the signals captured from the photodetector.

Our WT‐AFM methodology was conceived and refined to overcome the limitations of conventional frequency demodulation techniques. By employing advances in wavelet‐transform‐based AFM (WT‐AFM) modalities, we replace traditional bandwidth‐constrained LIA‐based systems with a continuous WT (CWT)‐based multiscale decomposition to overcome constrained temporal resolution, limited bandwidth and obscured material‐specific dynamic signatures. Applied directly to the high‐resolution, real‐time deflection signals from the AFM photodetector, this strategy enables decomposition in the time and frequency domains [36]. Unlike Fourier‐based methods or conventional LIAs, the wavelet‐transform approach preserves transient information and captures a broad spectral range rich in harmonic content, including both steady‐state and rapidly evolving non‐linear responses, with temporal resolutions reaching the microsecond scale (see Table 1 below) [32, 33, 34, 35]. It should be clarified that the WT‐AFM technique is inherently distinct from multi‐frequency AFM. Multi‐frequency AFM is a *physical excitation* technique, where the cantilever is simultaneously driven at multiple resonances to allow simultaneous topography and property mapping. In contrast, WT‐AFM is a *mathematical and computational analysis technique* applied to the resulting cantilever deflection signal.

**TABLE 1:** Comparison between WT‐AFM and LIA‐based and multi‐frequency AFM methods.

Feature	Traditional LIA	Multi‐frequency AFM	WT‐AFM
Frequency bandwidth	Narrow (*centred at cantilever resonance*, *ω*_0_)	Moderate (*limited to selected modes*)	Full (*broad*, *continuous frequency spectrum via CWT*)
Temporal resolution	Milliseconds	Variable (*typically tens to hundreds of microseconds*)	Microseconds (*with temporal resolution equal to that of the collected from original cantilever signal by DAQ*)
Transient detection	No	Partial (*averaged*, *filtered signals*)	Yes (*resolves non‐linear*, *short‐lived events*)
Feedback required	Yes (*for amplitude or frequency tracking*)	Yes (*e.g.*, *in intermodulation or bimodal modes*)	Yes (*open‐loop operation possible due to direct analysis of raw signal* *)*
Data processing and interpretation	Phase/amplitude *via* LIA	Phase/amplitude at selected frequencies	Phase/amplitude of all frequencies/harmonics; Wavelet transform coefficients + machine learning image fusion (AFM‐ICE)
System complexity	Low (*but limited capability*)	High (*requires multiple LIA channels and tuning*)	Moderate (*high computational load*, *simplified hardware*)
Noise sensitivity	Moderate–low (*depends on lock‐in time constants*)	Moderate–high (*more channels = more noise sources*)	Low (*wavelet transform filtering enables denoising without information loss*)

This perspective will outline a complete workflow incorporating high‐speed signal acquisition, WT‐based analysis, ML‐driven image enhancement and physics‐informed interpretation via digital twin simulations.

## 2 Fundamentals of WT‐AFM

A wavelet is a mathematical function defined as a short, rapidly decaying oscillation with finite energy, providing it with the crucial property of compact support, meaning it is localised in both time and frequency domains. The WT itself is the method used to decompose a given signal f(t), into a two‐dimensional representation or a scalogram, by correlating the signal with a family of the basis functions (called as daughter wavelets) generated from a single mother wavelet, ψ(t), through continuous or discrete scaling (corresponding to the frequency domain) and translation (corresponding to the time domain). Unlike the conventional Fourier transform, which uses infinite, non‐local sine waves and yields high‐frequency resolution but lacks time resolution, the WT uses localised wavelets to provide simultaneous time and frequency localisation, making it the superior mathematical tool for analysing non‐stationary signals characterised by transients, spikes or time‐varying frequencies similar to those observed in AFM.

It should be noted that in the context of WT‐AFM measurements, the choice of the mother wavelet, ψ(t), directly determines the quality and interpretability of the extracted data, particularly when analysing the non‐stationary, oscillatory cantilever deflection signal. As such, there is no single universally superior wavelet; rather, the selection must align the wavelet's mathematical characteristics with the specific analytical objective. For instance, a crucial factor is the wavelet's support, which is defined as its finite duration in the time domain. Wavelets with short, compact support, such as the Haar wavelet, excel at time localisation, making them ideal for identifying sharp discontinuities or abrupt changes in force at the instant of tip‐sample contact. However, for dynamic AFM analysis, the most significant decision involves managing the time–frequency trade‐off, which is governed by the ‘number of cycles (*n*)’ parameter within the widely used Morlet wavelet. A high *n* results in a longer support and better frequency resolution (suitable for resolving subtle shifts in the cantilever's resonant frequency, which may correlate with material stiffness or other material properties), but it sacrifices time resolution. Conversely, a low *n* value provides superior temporal resolution (essential for localising the exact time an event occurs on the microsecond scale) but yields poorer frequency resolution. Beyond this trade‐off, the wavelet's vanishing moments (related to its smoothness) are important for denoising and data compression, with wavelets possessing higher moments (e.g., Daubechies) providing a sparser representation of smooth data.

**FIGURE 1:**
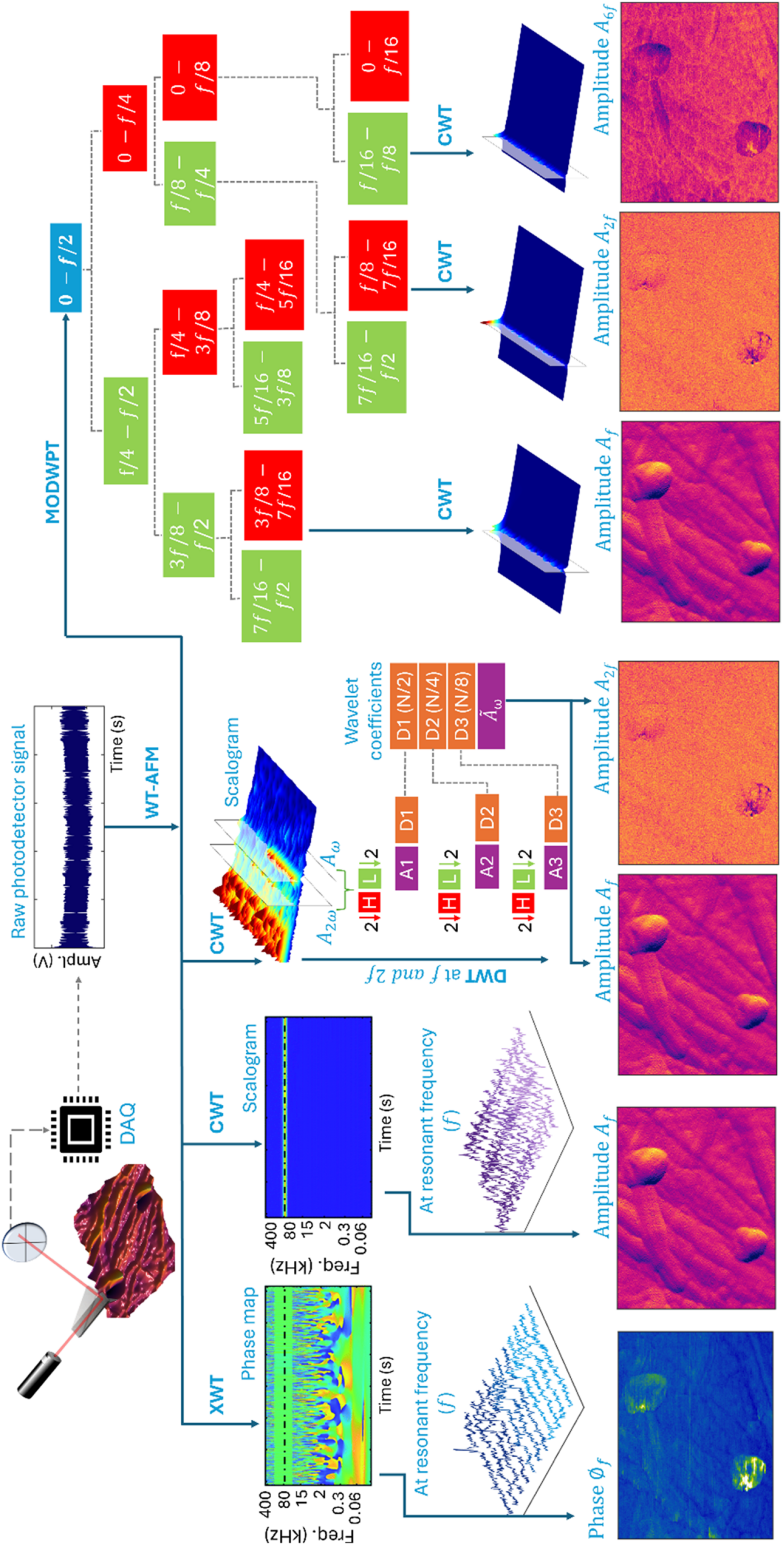
**S**chematic overview of the wavelet‐transform‐based AFM (WT‐AFM). The photodetector AFM cantilever signal is recorded by the DAQ and used for further WT analysis. The continuous wavelet transform (CWT) is the central analytical framework for the time–frequency localisation of AFM signals. The cross wavelet transform (XWT), derived from CWT, extracts the phase information between the cantilever and drive signal. The discrete wavelet transform (DWT) can be applied after CWT to refine harmonic amplitudes extraction by reducing fluctuations associated with CWT filter‐bank overlap. The maximal overlap discrete wavelet packet transform (MODWPT) may be used as a pre‐processing step before CWT to accurately capture amplitude information from higher‐order harmonics through recursive, non‐decimated filtering. Together, these WT‐processed outputs yield high‐resolution amplitude and phase maps of the sample under study as well as the harmonics and eigenmode‐resolved imaging channels.

The fundamental mechanism of the WT‐based AFM (WT‐AFM) method is illustrated in Figure 1. In this approach, the cantilever's vertical deflection signal is directly acquired in real time via a high‐speed Data Acquisition Board (DAQ). The raw deflection data are recorded at high temporal resolution (typically ≥1 MS/s) and high signal‐to‐noise ratio, from the photodetector without any pre‐filtering or demodulation, ensuring accurate preservation of transient, non‐linear and multi‐frequency responses across the entire frequency spectrum. It should be noted that the sampling rate (*f*_*s*_) must satisfy the Nyquist criterion ($f_{s}>2f_{\max})$ to avoid aliasing, which may lead the WT to interpret the signal at incorrect, coarser scale thus rendering any time‐resolved analysis of transient dynamics impossible. To practically realise this high‐speed digitisation without information loss, particularly when extracting weak, high‐frequency components, the DAQ hardware must meet rigorous performance benchmarks. For instance, the analogue front‐end requires a low input‐referred noise density ($\leq 30\;nV/\sqrt{Hz}$) to ensure small deflection variations are not masked by instrument electronics. While standard DAQs utilise nominal 16‐bit analogue‐to‐digital convertors (ADCs), the hardware implementation must maintain a high‐frequency effective number of bits (ENOB ≥ 12–14 bits) at full operational sampling (1–5 MS/s, or even higher) speed to achieve target harmonic SNRs exceeding ∼20–30 dB for robust MODWPT decomposition and maintain sufficient quantisation resolution for cantilever deflections. It is also worth mentioning that in our current workflow while the signal acquisition and streaming are performed in real‐time, the subsequent analysis workflow, including MODWPT processing, dimensionality reduction, clustering and image fusion, are currently implemented offline, with future real‐time deployment requiring dedicated FPGA‐ or GPU‐based acceleration.

The CWT is then applied to the acquired photodetector signal to obtain a multi‐dimensional representation encompassing amplitude, phase, frequency and time. Mathematically, the CWT of a time‐domain signal is defined as the convolution of the signal, *x*(*t*), with a localised wavelet, *ψ*(*t*), known as the mother wavelet. We have used the analytic Morse wavelet with parameters *γ *= 3 and *P* ^2^ = 60, where *γ* controls the symmetry and compactness of the wavelet in the frequency domain, while *P* ^2^ determines the time‐bandwidth product governing the trade‐off between temporal and frequency resolution [33]. These parameters provide balanced time–frequency localisation for the analysed AFM datasets:

(1) $$W\left(t,s\right)=\frac{1}{s}\int_{-\infty }^{\infty }x\left(u\right)\psi^{*}\left(\frac{u-t}{s}\right)du$$

where s is the scale parameter corresponding inversely to frequency, *t* is the temporal translation and * denotes the complex conjugate. The coefficients *W*(*t*,*s*) form a time–scale representation in which the magnitude corresponds to the instantaneous amplitude and the phase to the instantaneous phase. In this framework, the magnitude of the CWT coefficients represents the instantaneous amplitude, while the cross WT (XWT) between the cantilever deflection and drive signals captures phase relationships and shared oscillatory power (Supporting Information: Section 1). The resulting amplitude and phase maps (scalogram and phase map) reveal both transient and steady‐state dynamics, as shown in Figure 1. From these maps, amplitude and phase features can be extracted within the desired time–frequency intervals, enabling reconstruction of high‐resolution amplitude and phase images of the sample surface. The CWT adaptively balances time–frequency localisation: at low frequencies, longer wavelets provide superior frequency resolution, whereas at higher frequencies, shorter wavelets provide enhanced time resolution. This scale‐dependent flexibility enables precise quantification of instantaneous amplitude and phase variations in both harmonic and transient components of the signals. To minimise boundary effects in experiments, the wavelet footprint was adjusted to ensure that the wavelet duration exceeded the characteristic transient length of the recorded signal. The XWT extends this framework to analyse the interaction between two time series, typically, the cantilever deflection and drive signals, enabling simultaneous characterisation of shared spectral power and relative phase (Supporting Information: Section 1). XWT identifies time–frequency regions where the two signals exhibit coherent oscillatory behaviour, providing insights into phase synchronisation, locking or lagging dynamics between the excitation and response [32].

It is important to note that, while the CWT provides the central analytical framework for time–frequency localisation of AFM signal analysis, different applications necessitate the integration of additional signal processing or imaging methodologies to address specific experimental challenges. Depending on the modality, CWT needs to be complemented by other signal denoising and WT modalities to refine harmonic decomposition. Further, imaging approaches such as contrast analysis, clustering and image fusion can be used to systematically enhance interpretability (Supporting Information: Sections 2–4). These extensions are not independent of the CWT framework but build upon it, ensuring that transient nanoscale dynamics are first accurately localised in time and frequency and then further processed in a manner tailored to the particular experimental objectives.

For instance, the Maximal overlap discrete wavelet packet transform (MODWPT) [37] can be applied as a pre‐processing step prior to CWT analysis. MODWPT decomposes the photodetector signal into equal‐frequency sub‐bands, providing a noise‐robust representation with improved temporal resolution across the full spectral range. The CWT is then applied within each MODWPT sub‐band to extract amplitude information for both fundamental and higher‐order harmonics. By employing the redundancy of MODWPT alongside the precise time–frequency localisation of CWT, this approach enables systematic tracking of harmonic generation under varying tip–sample interaction conditions, as illustrated in Figure 1. MODWPT is able to provide uniform time–frequency resolution across all sub bands by recursively filtering both low‐ and high‐frequency components without down‐sampling, thereby preserving maximal spectral detail. However, this approach is computationally more demanding owing to its redundant and non‐decimated structure.

In contrast, the discrete WT (DWT) provides a compact, non‐redundant representation by recursively decomposing only the low‐frequency (approximation) components of the signal. While this makes the DWT computationally efficient, its sub‐bands have unequal bandwidths, which limits its ability to accurately resolve higher‐order harmonics. Consequently, the DWT primarily reflects an approximation of each sub‐band rather than providing detailed amplitude information at higher frequencies. Nevertheless, following CWT, the application of DWT with high‐order Daubechies wavelets (e.g., db45) can refine harmonic amplitude extraction, particularly for the second harmonic, where filter‐bank overlap in the CWT can introduce fluctuations, as demonstrated in Figure 1. More details of our WT analysis and approaches are given in the Supporting Information (Sections 5,6).

In the following sections, we highlight the principal applications of our WT‐AFM framework and illustrate its advantages through cases in which traditional LIA methods either impose substantial implementation complexity or fail to provide a viable solution.

## 3 WT‐AFM in Fundamental Study of Vibrational Harmonics

Understanding the fundamental mechanisms underlying vibrational harmonics at solid–solid interfaces is vital for advancing nanoscale material design and optimising mechanical and electronic performance [38, 39]. Interfacial vibrational harmonics are higher‐order frequency components generated when an AFM cantilever interacts with a surface through intrinsically non‐linear tip‐sample forces, and the behaviour of these interfacial vibrations governs key material properties, including stiffness, adhesion and energy dissipation by mediating complex surface interactions that bridge physics, chemistry and mechanics [40, 41]. Such interactions underpin technological innovations in nanotechnology, semiconductor engineering and biomaterials. Despite decades of progress in AFM technology, however, establishing direct correlations between measured cantilever vibration harmonics and underlying nanoscale physical parameters remains a major challenge. This difficulty primarily stems from the non‐linear nature of tip–sample interactions and the coexistence of attractive and repulsive forces, both of which induce harmonic generation and image contrast variations that are not yet fully understood [42, 43, 44].

Recent advances in WT‐AFM provide a transformative approach to overcoming these longstanding limitations. Unlike conventional amplitude modulation or multi‐frequency AFM, which typically rely on single/multiple‐frequency excitation and lock‐in detection, WT‐AFM enables real‐time, full‐spectrum decomposition of cantilever vibrations [34]. This approach captures transient frequency components, amplitude modulations and phase shifts arising from complex tip–sample dynamics. As a result, WT‐AFM allows the real‐time tracking of nanomechanical variations with sub‐millisecond temporal resolution, allowing direct observation of how material parameters such as elasticity, viscosity, adhesion and capillary forces govern harmonic behaviour [34]. By integrating this experimental framework with theoretical modelling, numerical simulations and digital twin–based data analytics, the origins of harmonic contrast and its dependence on specific material properties can be systematically explored [34]. Such insights deepen our understanding of interfacial nano‐mechanics and establish a foundation for designing advanced materials and AFM methodologies capable of quantifying complex mechanical responses with exceptional spatial and temporal precision.

**FIGURE 2:**
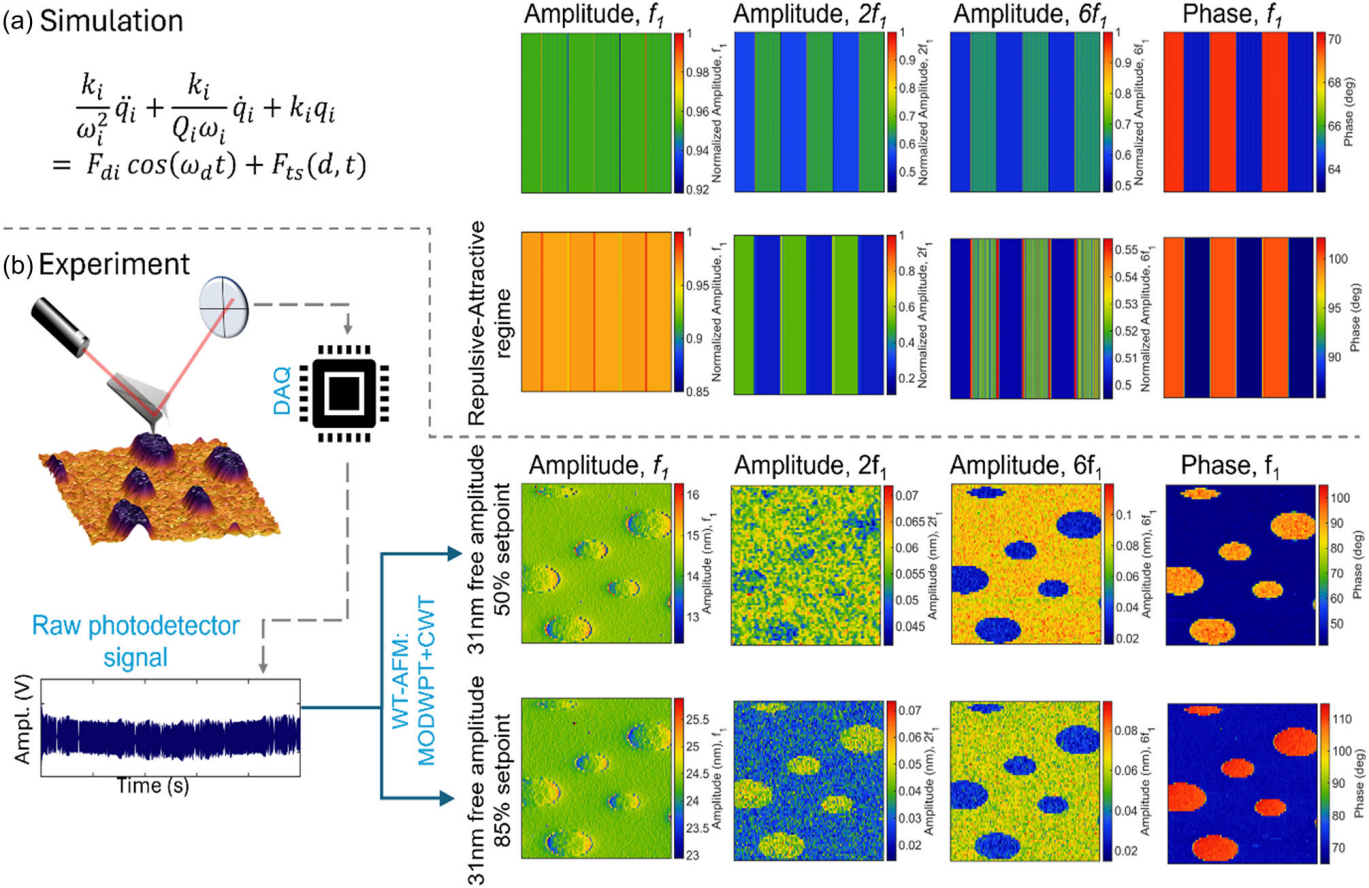
Application of WT‐AFM in studying vibrational harmonics at a solid–solid interface. (a) Simulations of the PS‐LDPE sample in both the repulsive and intermittent (repulsive‐attractive) regimes. The contrast reversal is observed in the intermittent regime due to the presence of both attractive and repulsive forces and the sensitivities of second and sixth harmonics to the attractive and repulsive forces, respectively. (b) Experimental WT‐AFM measurements on the PS‐LDPE sample with two different experimental settings. To extract amplitude information from higher‐order harmonics, the WT‐AFM framework is applied using a combined MODWPT and CWT analysis on the photodetector signal. At a 50% setpoint, the cantilever operates in the repulsive regime, and second and sixth harmonics display the same image contrast as the stiffness of the materials, reflecting the dominant role of sample stiffness in harmonic generation. At an 85% setpoint, the cantilever is in the intermittent interaction regime where both attractive and repulsive forces contribute to harmonics generation, which leads to the image contrast reversal between the second and sixth harmonics. As seen, there is an excellent agreement between simulation and experiments, which validate the hypothesis. In the simulation, the AFM parameters are chosen as $k=3.14\frac{N}{m},Q=223,f=76.6\;\mathrm{kHz}$ and R=10 nm. The physical parameters for PS are: Young's modulus 2.1±0.1 GPa, viscosity 418±100 Pa.s and Hamaker constant 50−100 zJ. For LDPE: Young's modulus 0.11±0.02 GPa, viscosity 38.5±1.5 Pa.s and Hamaker constant 50−100 zJ. More detailed information of simulation and experiments is given in Biglarbeigi et al. [34]. The figure is adopted with permission from [34] 2025, John Wiley & Sons Inc.

In our study, WT‐AFM was applied to four representative solid–solid interfaces, representing discontinuous boundaries, non‐linear viscoelastic behaviour and critical nanoscale heterogeneity, to investigate how harmonic responses depend on key mechanical and surface parameters [34]. For this, we examined samples, including polystyrene–low density polyethylene (PS–LDPE), polystyrene–poly(methyl methacrylate) (PS–PMMA) and Au nanoparticles deposited on highly ordered pyrolytic graphite (HOPG), as well as graphene oxide (GO) deposited on HOPG. Measurements on PS–LDPE and PS–PMMA were performed across a range of free and setpoint amplitudes to evaluate the harmonic behaviour under distinct mechanical and viscoelastic conditions. Figure 2 illustrates the schematic of the experimental methodology and shows both simulation and experimental representative results for the PS–LDPE system.

Our findings demonstrate that harmonics in close proximity to the second eigenfrequency of the microcantilever exhibit enhanced sensitivity to variations in the sample's stiffness. In addition, during surface scanning, we resolve two distinct interaction regimes, repulsive and intermittent, which give rise to a pronounced contrast reversal amongst the detected harmonics. Notably, our analysis reveals the previously overlooked influence of long‐range attractive interactions, particularly van der Waals forces, in the generation of higher‐order harmonics. Although often omitted in conventional interpretations of microcantilever‐based AFM measurements, these interactions are shown to play a crucial role in the observed vibrational response. By integrating these observations, our study offers new insights into the intricate dynamics of solid–solid interactions at the nanoscale, thereby advancing the fundamental understanding of tip–sample physics and informing the development of next‐generation nanomechanical characterisation techniques.

Since capturing the full range of solid–solid interactions requires the high‐frequency harmonics of the photodetector signal, we applied the MODWPT followed by CWT for detailed time–frequency analysis. Further, to quantify the resolution and contrast of the reconstructed harmonic images, objective quality metrics can be employed, including peak signal‐to‐noise ratio (PSNR), structural similarity index measure (SSIM) and inter‐image correlation (Supporting Information: Section 3) [34]. These metrics provide a rigorous basis for assessing how tip‐sample interactions, viscoelastic dissipation and capillary contributions influenced harmonic generation and propagation. This combined MODWPT‐CWT framework thus enables detailed characterisation of non‐linear vibrational dynamics at the interface, offering insights into how higher‐order harmonic content encodes material properties such as viscoelasticity, adhesion and surface energy at the nanoscale.

## 4 Multilayer and Heterogeneous Nanomaterials Imaging

The rapid advancement of AFM has revolutionised nanoscale characterisation, enabling molecular‐ and atomic‐level interrogation of increasingly complex material systems [45]. However, the increasing size and complexity of AFM datasets, particularly in multi‐layer and heterogeneous nanostructures, pose substantial challenges for extracting meaningful physical and chemical insights [45]. Conventional imaging and contrast enhancement methods often fail to resolve intricate interfacial details or differentiate between overlapping layers, embedded defects or compositional variations. These limitations are further compounded by the non‐linear tip–sample interactions that generate higher‐order harmonic oscillations in AFM signals. Unlike conventional topography or phase imaging, which primarily capture surface height or energy dissipation, harmonic signals encode rich information about nanoscale mechanical properties such as elasticity, adhesion and viscoelasticity [35]. A careful analysis of these harmonic components can transform AFM from a primarily morphological probe into a platform capable of mapping nanoscale variations in stiffness and composition, advancing the understanding of complex, layered materials.

**FIGURE 3:**
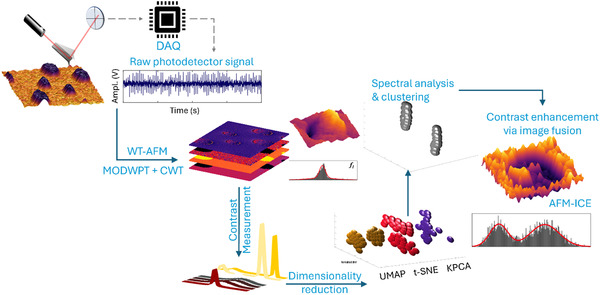
Schematic of the AFM image contrast enhancement (AFM‐ICE) pipeline within the WT‐AFM framework. The workflow involves high‐speed data acquisition of the photodetector signal, followed by extraction of higher harmonics using the combined MODWPT + CWT method, which isolates harmonics at both driven and non‐driven frequencies. Normalised harmonic amplitudes are then computed by mixing image products at driven and non‐driven frequencies to obtain contrast measures. These contrast measures are then visualised in multiple forms and subjected to spectral analysis, followed by clustering to interpret the clusters and their associated material properties, and finally, image fusion is employed for overall contrast enhancement [35]. The figure is adopted with permission from [35] 2025, John Wiley & Sons Inc.

**FIGURE 4:**
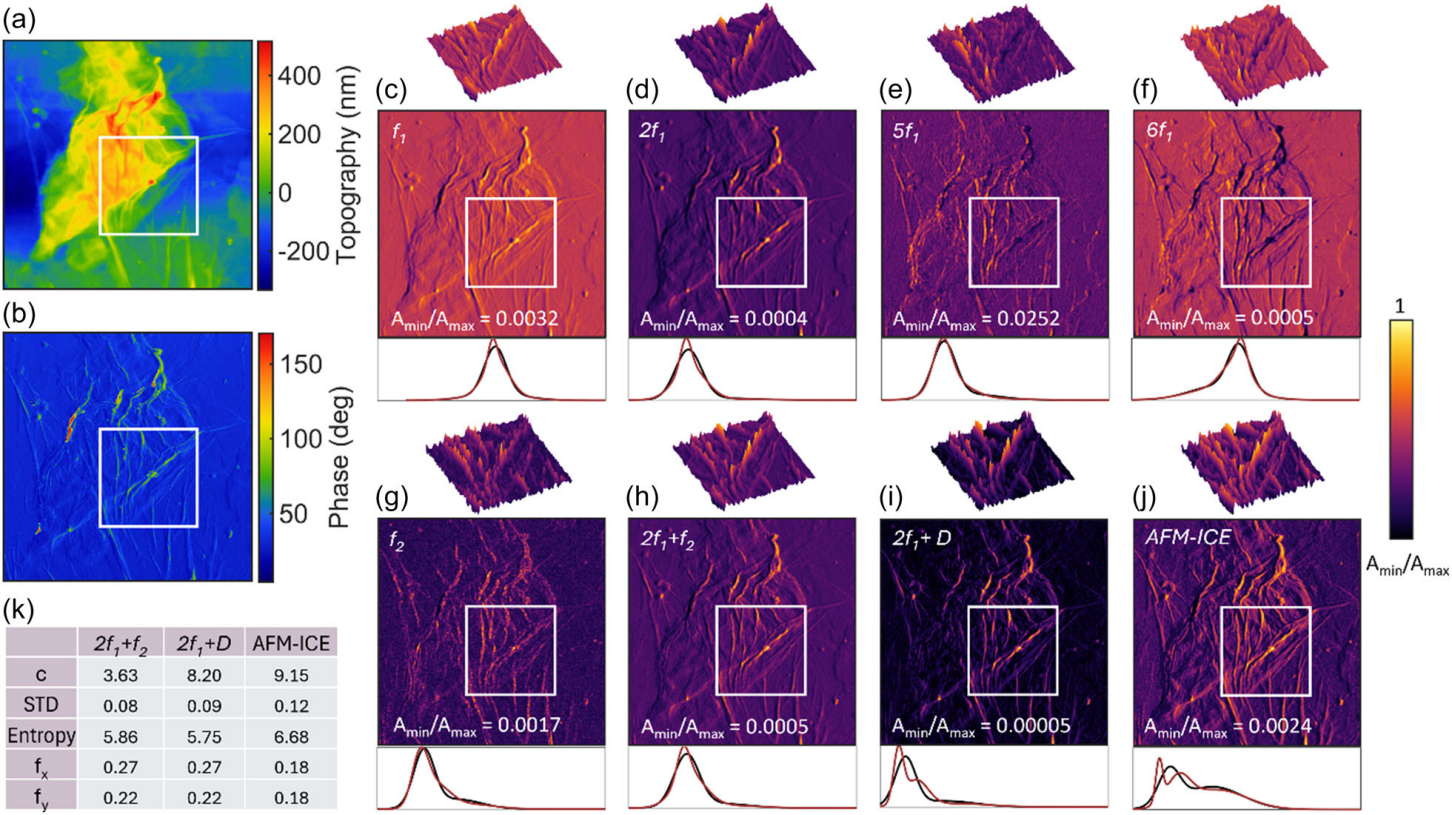
AFM‐ICE‐led analysis of Au nanoparticles deposited on a multi‐layer GO‐HOPG sample. An area of 12 × 12 µm of the sample was investigated. (a) Topography and (b) phase images of the sample were obtained via the conventional LIA‐based imaging. (c–j) The harmonic figures further represent normalised amplitudes obtained from the WT‐AFM method. A region of interest (ROI), shown with a box, of the sample is chosen to represent more details of the amplitude histogram (bottom panel), together with the zoomed‐in three‐dimensional figure (top panel) of the ROI representing the amplitude in both colour and *z*‐axis. The histogram shown in BLACK represents the summation of two fitted normalised histograms, corresponding to a two‐component assumption in which the GO and HOPG regions are treated as a combined background component relative to the Au nanoparticles. The histogram shown in RED represents the summation of three fitted normalised histograms, corresponding to full separation of the Au, GO and HOPG components. Comparison between the two‐ and three‐component fits demonstrates the enhanced material discrimination capability of AFM‐ICE, enabling clearer separation of overlapping compositional distributions within the heterogeneous multilayer structure. (k) Contrast measures and spatial frequency analysis for the selected images from the effective cluster, along with the enhanced image produced by the AFM‐ICE methodology [35], demonstrating the superior material discrimination and contrast enhancement. Here, c is the image contrast metric, STD is the standard deviation of image intensity, entropy represents the image information content and $f_{x}$ and $f_{y}$ denote the spatial frequency components along the horizontal and vertical directions, respectively. In this approach, we used the sample contrast index, image standard deviation, entropy as well as the images spatial frequency [35]. The figure is adopted with permission from [35] 2025, John Wiley & Sons, Inc.

To overcome these limitations, we developed a WT‐based framework integrated with unsupervised image processing and fusion algorithms**,** termed AFM Image Contrast Enhancement (AFM‐ICE) [35]. This method enhances image contrast by isolating material‐specific features within complex nanoscale environments by simultaneously measuring multiple harmonics and frequencies from a single cantilever scan without the need for additional hardware or excitation of multiple eigenmodes. By exploiting the full harmonic spectrum embedded in the cantilever signal, AFM‐ICE substantially improves the interpretability of multi‐layer systems composed of interfacial heterogeneities, defects and deposited nanoparticles. The methodology demonstrates superior performance in differentiating components of examined heterogenous materials, revealing subtle mechanical and structural contrasts within layered architectures [35]. This approach represents a significant advance towards rapid, high‐fidelity and physics‐informed AFM imaging for heterogeneous materials. The schematic of the proposed WT‐based AFM combined with the AFM‐ICE workflow is presented in Figure 3, along with representative results from the Au nanoparticles–GO–HOPG system in Figure 4.

In this approach, dynamic‐mode AFM experiments were conducted by exciting the cantilever at its first resonance frequency while continuously recording the response signal. Using WT‐based techniques, the time–frequency decomposition of the signal was obtained, allowing the reconstruction of amplitude and phase images corresponding to the excitation frequency (*f*_1_), second eigenfrequency (*f*_2_), second through to sixth harmonics and their non‐linear mixing products. The WT‐AFM data provide high temporal and spatial resolution, determined by the sampling frequency of the DAQ system, effectively capturing transient dynamics down to the microsecond scale. To allow a measurable comparison with standard topography and phase images, all images (*n* = 30) were rescaled to 256 × 256 pixels by averaging the neighbouring data points along the oversampled scan axis, thereby reducing data density while preserving the overall spatial features and contrast of the original images. Multiple image contrast metrics, including correlation, standard deviation and entropy, were then calculated as explained in Biglarbeigi et al. [35]. Given the high dimensionality of the dataset and non‐linear material response, we evaluated multiple non‐linear embedding methods, including kPCA (Gaussian and Laplacian kernels), t‐SNE and UMAP (Euclidean, cosine, and correlation distances). To ensure diversity of representations, eight parameter scenarios were defined *a priori* to span different assumptions in kernel structure, neighbourhood geometry and manifold embedding behaviour. Rather than performing optimisation towards a single objective function, these configurations were used to generate a set of complementary embeddings for downstream spectral meta‐visualisation, consistent with the framework of Ma et al. [46].

This was followed by unsupervised ML clustering to define the effective and non‐effective clusters. Cluster interpretation was performed without ground‐truth labels. In the absence of ground‐truth labels, and as is standard in unsupervised clustering analyses, cluster interpretation was performed post hoc using image‐quality metrics. As such, cluster quality was assessed using image‐based contrast metrics, including contrast index, standard deviation, entropy and spatial frequency measures, where configurations yielding consistently higher separability across these measures were designated as 'effective' for fusion analysis.

Consequently, images belonging to the effective cluster displayed well‐defined structural and compositional features. Fusing two normalised images from this cluster yielded enhanced representations containing greater quantifiable information about material composition and interfacial structure. It is worth noting that although the WT‐AFM acquisition is performed in real‐time through high‐speed DAQ streaming, the complete AFM‐ICE workflow shown in Figure 3 is currently implemented as an offline post‐processing pipeline.

Therefore, this workflow uses unsupervised ML combined with systematic hyperparameter screening across embedding methods. The parameter scenarios are not dataset‐specific and are intended to provide general embedding diversity; however, their relative performance may vary across different material systems and therefore, may require reassessment when applied to new datasets.

To evaluate the performance of AFM‐ICE, we examined four sample systems: (I) SWCNTs deposited on a silicon substrate with a native oxide layer, (II) multilayer GO‐HOPG, (III) Au nanoparticles deposited on multilayer HOPG and (IV) Au nanoparticles deposited on multilayer GO‐HOPG. Across all sample types, the proposed methodology effectively captured the fine structural and compositional differences. For SWCNTs, AFM‐ICE enhanced contrast, eliminated topography artefacts caused by feedback errors and accurately resolved nanotube dimensions [35]. In the GO‐HOPG sample, the technique clearly differentiated between GO and HOPG layers, enabling precise quantification of layer proportions. For Au nanoparticles on multi‐layer HOPG, the approach facilitated accurate characterisation of nanoparticle spatial distribution [35]. Notably, in the Au nanoparticle–GO–HOPG sample (Figure 4), the method successfully discriminated among three distinct components, Au, GO and HOPG, a task that is challenging for conventional image analysis methods or post‐processing techniques. To validate robustness, the AFM‐ICE approach was applied across multiple regions and scan sizes for each sample. Statistical analyses [35] (Figure 4) confirmed significant improvements in image contrast and material discrimination across all heterogeneous systems studied. Histogram‐based probability density function (PDF) analysis further demonstrated that AFM‐ICE enhances the separability of component distributions by improving histogram peak resolution, enabling accurate quantification of material composition. Enhanced contrast through image fusion yielded higher values of contrast, standard deviation and entropy, indicating more detailed and information‐rich images.

## 5 Time‐Resolved Kelvin Probe Force Microscopy (KPFM)

Understanding nanoscale charge generation, separation and transport is fundamental for designing the next‐generation functional materials and devices such as perovskite photovoltaics, photocatalysts, energy harvesters and quantum systems [33]. Conventional KPFM has been invaluable for mapping SP and contact potential difference (CPD), yet its feedback‐based operation imposes intrinsic temporal limitations [47], wherein the LIA averages transient signals and suppresses higher‐order harmonics, obscuring sub‐millisecond electrostatic processes that underpin real‐time charge dynamics. Consequently, the conventional closed‐loop KPFM (CL‐KPFM) fails to capture the non‐stationary charge kinetics crucial for understanding fast interfacial phenomena.

**FIGURE 5:**
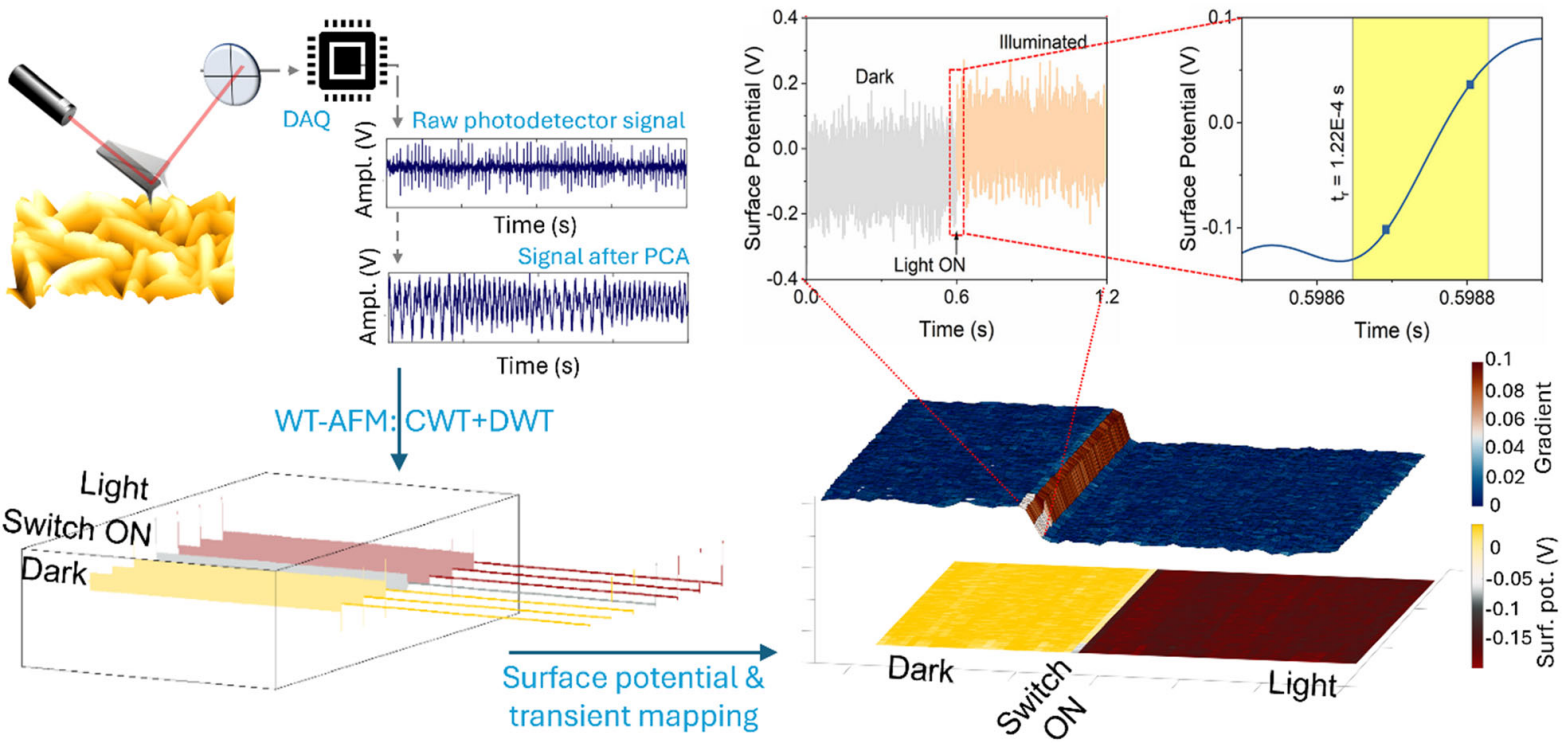
Schematic of the proposed time‐resolved OL‐WT‐KPFM framework. The cantilever photodetector signal is acquired by DAQ and processed through the CWT, DWT and PCA framework. PCA denoises the raw time‐domain data, while the combined CWT and DWT extract the instantaneous amplitudes of the first and second harmonics, as well as the phase. These quantities are then used to compute the surface potential (SP), capacitance gradient and dielectric constant of materials. The OL‐WT‐KPFM mapping, with a temporal resolution of 1 μs, enables probing of both the steady‐state SP response and fast dynamic processes. The method captures the switching ON surface photovoltage transient (across the line, enclosed by the red dashed box; zoomed into the right inset) in the photoactive BiOI semiconductor yielding an extracted rise time of ∼112 μs, with a slope of 1.22 mV/μs [33]. The figure is adopted with permission from [33] 2023, American Chemical Society.

To address these limitations, we developed a WT‐based, open‐loop KPFM (OL‐WT‐KPFM) method, illustrated in Figure 5, which achieves microsecond temporal resolution, nearly three orders of magnitude faster than conventional approaches. This feedback‐free approach integrates open‐loop signal acquisition with CWT analysis, enabling simultaneous quantification of SP, capacitance gradient and dielectric constant from a single photodetector data stream. Unlike previously reported systems requiring multiple LIAs, the OL‐WT‐KPFM reconstructs the full spatiotemporal evolution of the cantilever motion, capturing non‐linearities and higher harmonics without any feedback‐induced averaging. In this technique, the analysis begins with denoising of the raw photodetector signal using PCA, as shown in Figure 5. This step is particularly effective in experiments involving repeated transient patterns, such as pulsed excitations arising from chopped illumination or electrical input, where consistent features can be retained while uncorrelated noise is suppressed. To perform this step, the deflection signal is organised into a two‐dimensional data matrix in which each column represents an individual pulse (corresponding to the input electrical pulse). Singular value decomposition (SVD) is then applied to identify the principal components of the dataset (Supporting Information: Section 2). The components associated with the largest singular values are then retained to reconstruct the denoised signal, whereas those with smaller singular values, based on the 'elbow' criterion from the scree plot, are excluded as noise [33]. It should be noted that unlike traditional LIAs that suppress noise via continuous temporal integration, that invariably smears fast transient events, the PCA/SVD orthogonal subspace projection isolates the deterministic signal (high variance leading principal components) from stochastic noise (low variance trailing components) in the statistical variance domain, allowing for robust noise suppression while preserving the full temporal sampling density of the original transient waveform.

Importantly, the PCA/SVD denoising procedure employed here assumes that the transient response is reproducible across repeated excitation cycles. The method exploits ensemble statistics obtained from multiple measurements of the same physical event, retaining waveform features that are consistent from pulse to pulse while suppressing uncorrelated noise. Consequently, this denoising strategy is most effective for repetitive transient phenomena, such as periodically pulsed optical or electrical excitations. For fully non‐repetitive single‐shot events, where no ensemble of repeated responses exists, PCA/SVD denoising cannot be applied in its present form.

This limitation, however, applies only to the denoising stage and not to the wavelet‐transform framework itself. The WT‐based analysis operates directly on individual time‐domain signals and can therefore be applied to arbitrary single‐shot transients, provided that the acquired signal‐to‐noise ratio is sufficient for reliable extraction of the relevant harmonic or transient components. In such cases, the raw or conventionally filtered photodetector signal can be analysed directly using the continuous and discrete WTs without requiring ensemble averaging.

It is also important to distinguish this ensemble‐based denoising from the temporal averaging performed by conventional LIAs. In LIA‐based detection, noise suppression is achieved through narrow‐band filtering and temporal integration, which inherently reduces measurement bandwidth and attenuates fast transient components and higher harmonics. By contrast, the PCA/SVD approach suppresses noise across an ensemble of repeated measurements while preserving the complete temporal waveform acquired at the DAQ sampling rate. Thus, whereas LIA averaging fundamentally trades temporal resolution for signal‐to‐noise ratio, PCA/SVD denoising preserves the intrinsic temporal bandwidth of the transient response but assumes repeatability between excitation cycles.

Accordingly, the two approaches are applicable under different experimental conditions. LIA detection is well suited for stationary or periodically modulated signals where bandwidth reduction is acceptable and maximum sensitivity is required. The PCA/SVD‐enhanced OL‐WT‐KPFM methodology is particularly advantageous for repetitive transient processes where both high signal‐to‐noise ratio and preservation of waveform dynamics are essential. For arbitrary non‐repetitive single‐shot events, the WT framework remains applicable without PCA/SVD denoising, enabling time–frequency analysis of transient electrostatic phenomena while retaining the full measurement bandwidth.

Following denoising, CWT is then applied to extract the instantaneous amplitudes of the first $\left(A_{f}\right)$ and second ($A_{2f}$) harmonics, which are required to calculate the CPD ($V_{\mathrm{CPD}}$) using the following equation:

(2) $$V_{\mathrm{CPD}}=\frac{A_{f}\cos(\phi_{f})}{A_{2f}}\frac{V_{\mathrm{AC}}}{4X_{\mathrm{gain}}}$$

where $V_{\mathrm{AC}}$ is the applied AC drive signal, $\phi_{f}$ represents the phase between the cantilever response and the applied drive signal and $X_{\mathrm{gain}}=G(f)/G(2f)$ is the cantilever transfer function.

Here, the first and second harmonic amplitudes are governed by:

(3) $$A_{f}=G\left(f\right)\frac{\left|F_{f}\right|}{k}=G\left(f\right)|\frac{\partial C}{\partial z}V_{CPD}|\frac{V_{AC}}{k}$$

(4) $$A_{2f}=G\left(2f\right)\frac{\left|F_{2f}\right|}{k}=G\left(2f\right)|\frac{\partial C}{\partial z}|\frac{V_{AC}^{2}}{4k}$$

where *∂C*/*∂z* is the local capacitance gradient. Substituting these expressions into Equation (2) reveals that the capacitance‐gradient term cancels identically during reconstruction of *V*_CPD_. Consequently, local variations in tip–sample capacitance arising from surface roughness, curvature or other geometric factors are mathematically removed on a pixel‐by‐pixel basis, ensuring that the reconstructed surface‐potential map reflects only the true electrostatic potential. While capacitance‐gradient maps are expected to correlate with topography because *∂C*/*∂z* is inherently governed by local geometry, this geometric contribution does not propagate into the final *V*_CPD_ reconstruction. It should be noted that all OL‐WT‐KPFM measurements were performed in the dual‐pass lift‐mode operation. During the first pass, the sample topography was acquired using conventional tapping‐mode AFM, while during the second pass, the tip retraced the recorded topography at a constant lift height (∼50 nm). This measurement geometry minimises direct topography‐feedback coupling and allows electrostatic interactions to be measured under well‐defined tip‐sample separation conditions.

Following CWT, the DWT with high‐order Daubechies wavelets (db45) is employed to refine the extraction of harmonic amplitudes, particularly for the second harmonic, where filter‐bank overlap in the CWT can introduce fluctuations. Phase information is extracted by performing XWT between the photodetector signal and the drive signal (Supporting Information: Section 1), yielding the local phase lag of the first harmonic. Together, the harmonic amplitudes and phases provide a complete basis for the quantitative determination of SP, as illustrated in Figure 5. In particular, the CPD can be calculated from the ratio of the first and second harmonic contributions, allowing nanoscale mapping of work function and dielectric variations with high temporal resolution.

To experimentally validate the separation between electrostatic and geometric contributions, a standard EFM‐KPFM calibration sample comprising lithographically patterned Au and Al lines on an oxide‐covered Si substrate was also investigated (Figure S1). Mapping of the localised second harmonic (*A*_2_ *_f_*) successfully reconstructed quantitative capacitance‐gradient (*∂C*/*∂z*) variations, with enhanced signals observed at metallic boundaries owing to geometric edge effects and increased capacitive coupling between the AFM tip cone and the sidewalls of the metallic structures. These measurements confirm that OL‐WT‐KPFM explicitly resolves geometric capacitance variations through the *∂C*/*∂z* channel while maintaining independent reconstruction of the surface‐potential signal.

**FIGURE 6:**
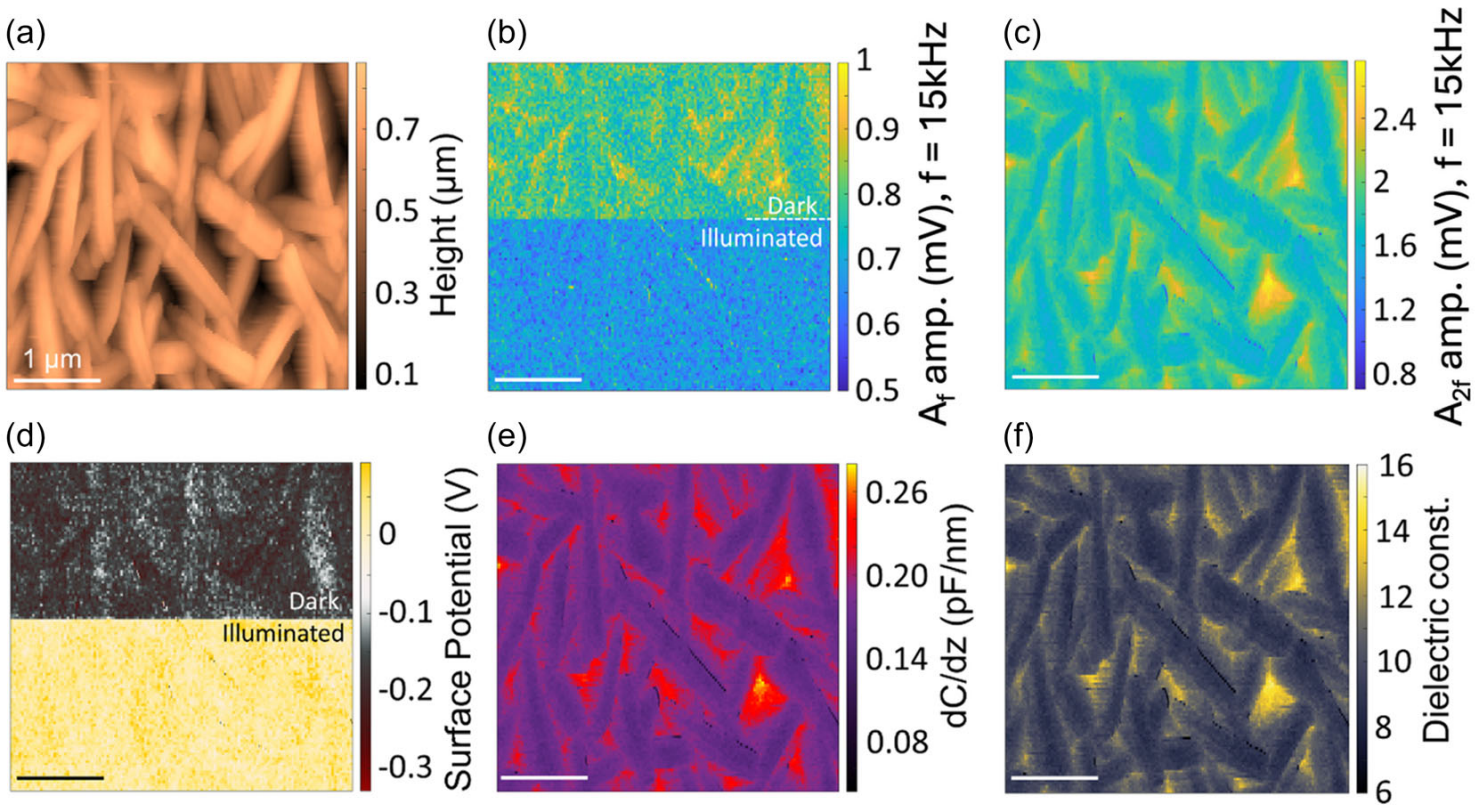
Imaging and information extraction capabilities of the OL‐WT‐KPFM technique. (a) Topography of the *n*‐type BiOI sample. Using the photodetector signal, the CWT enables extraction of (b) the first harmonic (*A*_*f*_), (c) the second harmonic (*A*_2*f*_) and the corresponding (d) surface potential map. (e) The capacitance gradient (*∂C/∂z*) mapping and (f) the corresponding dielectric constant (*ε*) values were also computed for the sample. The dotted line in (b) represents the switching ON of the illumination to the sample. The horizontal scale bar represents 1 μm [33]. The figure is adopted with permission from [33], 2023, American Chemical Society.

Using bismuth oxyiodide (BiOI) as a model semiconductor, OL‐WT‐KPFM successfully resolved illumination‐induced surface photovoltage (SPV) transients with the reported values comparable to those measured using the bulk surface photovoltage technique, revealing charge diffusion and recombination dynamics that are inaccessible to conventional closed‐loop based KPFM (Figure 5). The CWT‐derived maps of SP, capacitance gradient and dielectric constant showed excellent consistency with conventional measurements, while offering greater temporal insight into charge carrier motion under optical excitation (Figure 6). Complementary simulations performed across cantilevers spanning a range of resonance frequencies verified the accuracy of the reconstructed interaction potentials for transient events as short as 10 μs and established an ultimate temporal detection limit of approximately 200 ns, a simulation‐bounded theoretical limit determined by the combined constraints of DAQ bandwidth and cantilever transfer‐function dynamics [33].

This OL‐WT‐KPFM approach bridges the temporal gap between electronic excitation and charge relaxation, providing a comprehensive platform for quantifying transient electrostatic behaviour in functional materials. By combining the spatial resolution of AFM with WT‐based time–frequency analysis, OL‐WT‐KPFM enables direct visualisation of dynamic charge processes, establishing a new paradigm for nanoscale probing of ultrafast interfacial phenomena far beyond the reach of traditional KPFM modalities.

## 6 Discussions and Outlook

Recent advances in WT‐AFM are reshaping the landscape of nanoscale imaging and spectroscopy. By bypassing the traditional LIAs and feedback controllers, adopting an open‐loop configuration and integrating advanced computational and unsupervised learning algorithms, the suite of WT‐AFM techniques unlocks the full dynamic potential of cantilever responses. The simultaneous acquisition of the complete spectral content of the photodetector signal enables access to all harmonics, sub‐harmonics and non‐integral frequency components without any additional hardware. This multi‐dimensional dataset provides unprecedented insight into the mechanical, physical, chemical and electronic properties of materials, transforming AFM into a quantitative, time‐resolved microscopic and spectroscopic platform.

The AFM‐ICE framework exemplifies the power of this approach [35]. By extracting the non‐linear harmonic responses of the cantilever in a single scan, AFM‐ICE significantly improves image contrast and enhances material discrimination, particularly in heterogeneous and multilayer systems. WT‐based image fusion and unsupervised learning further refine data quality, allowing autonomous detection of nanoscale variations in composition, structure and morphology. Unlike conventional multi‐frequency AFM approaches, which require multiple eigenmode excitations and external lock‐ins, AFM‐ICE extracts rich contrast information directly from intrinsic surface–cantilever interactions. This approach not only enhances interpretability but also ensures scalability and automation for high‐throughput materials characterisation. While AFM‐ICE provides a major step forward in nanoscale imaging, the computational demand associated with integrating multiple visualisation and fusion techniques remains high, particularly for large WT‐AFM datasets. Future efforts will focus on optimising computational efficiency through parallel processing, high‐performance computing resources and the incorporation of advanced ML algorithms for spectral analysis. The integration of deep learning frameworks, such as convolutional neural networks, could enable automated, adaptive enhancement and material discrimination, extending AFM‐ICE's applicability to sharper features and tip–sample deconvolution challenges. Moreover, combining AFM‐ICE with model‐based harmonic analysis would link image contrast to physical quantities such as Young's modulus and viscosity, preserving the mechanical interpretability of the fused images.

Similarly, the OL‐WT‐KPFM introduces transformative advances over traditional time‐resolved KPFM methods [33]. Unlike pump–probe [48, 49] or heterodyne time‐resolved KPFM [49], which are limited by synchronisation constraints, cantilever bandwidth and feedback‐loop assumptions, OL‐WT‐KPFM captures transient SP changes directly and continuously. The use of localised WT basis functions preserves non‐stationary and short‐lived electrostatic features that are typically lost in other time‐resolved KPFM modes such as G‐mode or F3R‐KPFM [47] due to temporal averaging. By eliminating the need for LIAs, feedback biasing and model‐dependent deconvolution, OL‐WT‐KPFM simplifies implementation while improving reproducibility and robustness across diverse material systems. This enables quantitative, microsecond‐resolved measurement of charge carrier dynamics and electrostatic relaxation processes, providing direct access to physical mechanisms previously hidden by feedback‐induced distortions. Although transfer‐function modelling indicates a simulation‐bounded theoretical temporal limit approaching ∼200 ns, the shortest transient experimentally resolved to date remains ∼112 μs, highlighting the need for future cantilever‐response deconvolution frameworks and bandwidth‐engineering strategies to bridge the gap between theoretical and experimentally accessible time resolution.

Collectively, WT‐AFM establishes a unified framework for real‐time, high‐resolution analysis of transient nanoscale phenomena across broad frequency and temporal ranges. It provides a platform that merges experimental precision with computational intelligence, bridging the gap between mechanical response, electronic structure and material functionality. At present, the real‐time capability primarily applies to high‐speed signal acquisition and streaming, whereas advanced AFM‐ICE operations such as MODWPT processing, dimensionality reduction, clustering and image fusion are performed offline and will require FPGA‐ or GPU‐accelerated implementations for fully real‐time deployment.

Despite these significant advantages, several technical challenges remain. The high‐dimensional nature of WT decomposition and machine‐learning‐based analysis demands considerable computational resources and algorithmic optimisation to enable efficient real‐time processing. High‐bandwidth data acquisition produces extensive datasets, necessitating reliable data storage, management and compression strategies. Real‐time analysis and adaptive feedback control may require the deployment of specialised hardware such as field‐programmable gate arrays (FPGAs) or GPU‐accelerated processors. Moreover, the accurate reconstruction and interpretation of multi‐frequency harmonic data call for advanced expertise in both signal processing and nanoscale mechanics. Rigorous data preprocessing is equally critical to minimise errors arising from thermal drift, parasitic couplings and scanner non‐linearities, which can compromise the accuracy of WT reconstructions and physical parameter extraction.

Future work will focus on employing ML and advanced image processing to overcome these challenges and unlock the full potential of WT‐AFM. Deep learning architectures, particularly convolutional and graph‐based neural networks, will be employed to automate pattern recognition, feature extraction and dynamic mode classification in multi‐dimensional AFM datasets. Real‐time AI‐assisted feedback mechanisms will enable autonomous tuning of experimental parameters, ensuring adaptive optimisation during scanning. The integration of ML‐driven image enhancement with digital twin simulations will further support physics‐informed data interpretation, offering predictive insights into nanoscale processes rather than purely descriptive measurements. Parallel advancements in on‐chip data processing, high‐speed acquisition systems and AI‐accelerated analytics are anticipated to make WT‐AFM more accessible, efficient and scalable.

Ultimately, the convergence of open‐loop AFM architectures, AI‐driven analytics and computational modelling will transform WT‐AFM from a descriptive imaging technique into a predictive, data‐rich and autonomous nanoscale analysis platform. Such a transformation will significantly accelerate discovery and innovation across diverse fields, including nanomaterials science, quantum device engineering, energy conversion systems and biological interface characterisation, ushering in a new era of intelligent and self‐optimising AFM.

## Funding

This work was supported by National Institute for Health and Care Research (NIHR303160), UK Research and Innovation (FLF Fellowship UKRI3091) and the Department for the Economy (DfE), Northern Ireland (USI 186).

## Conflicts of Interest

The authors declare no conflicts of interest.

## Supporting information

Supplementary Material

## Data Availability

The data that support the findings of this study are available from the corresponding author upon reasonable request.

## References

[1] A. Johnston and E. H. Sargent , “Accelerated Discovery of Optoelectronic Materials,” ACS Photonics 8 (2021): 699–701.

[2] R. Omar , J. Yang , T.‐P. Huynh , et al., “Advanced Materials in Responsible Electronics: Innovations for Sustainability, Health, and Circularity,” Advanced Materials Technologies 10, no. 23 (2025): e01020, 10.1002/admt.202501020.

[3] C. Lin , A. Consiglio , O. K. Forslund , et al., “Uniaxial Strain Tuning of Charge Modulation and Singularity in a Kagome Superconductor,” Nature Communications 15 (2024): 10466. 10.1038/s41467-024-53737-w PMC11612471 39622808

[4] M. Jakešová , M. Silverå Ejneby , V. Đerek , et al., “Optoelectronic Control of Single Cells Using Organic Photocapacitors,” Science Advances 5, no. 4 (2019): eaav5265. 30972364 10.1126/sciadv.aav5265 PMC6450690

[5] P. Sun , C. Li , C. Yang , et al., “A Biodegradable and Flexible Neural Interface for Transdermal Optoelectronic Modulation and Regeneration of Peripheral Nerves,” Nature Communications 15 (2024): 4721. 10.1038/s41467-024-49166-4 PMC11148186 38830884

[6] J. Han , K. Park , S. Tan , et al., “Perovskite Solar Cells.,” Nature Reviews Methods Primers 5 no. 3 (2025): 3.

[7] P. Kaifosh , T. R. Reardon , C. Anderson , et al., “A Generic Non‐Invasive Neuromotor Interface for Human‐Computer Interaction,” Nature 645 (2025): 702–711. 40702190 10.1038/s41586-025-09255-w PMC12443603

[8] Z. Hu , X. Liu , P. L. Hernández‐Martínez , et al., “Interfacial Charge and Energy Transfer in Van der Waals Heterojunctions,” InfoMat 4 (2022): e12290.

[9] Y. Zhou , J. Zhang , G. Ren , B. Y. Zhang , and J. Z. Ou , “Illuminating Quantum Phenomena in 2D Materials: The Power of Optical Spectroscopy,” Advanced Optical Materials 13, no. 31 (2025): e01714, 10.1002/adom.202501714.

[10] V. G. Kravets , A. V. Kabashin , W. L. Barnes , and A. N. P. S. Grigorenko , “Lattice Resonances: A Review of Properties and Applications,” Chemical Reviews 118 (2018): 5912–5951. 29863344 10.1021/acs.chemrev.8b00243 PMC6026846

[11] R. Garcia , “Nanomechanical Mapping of Soft Materials with the Atomic Force Microscope: Methods, Theory and Applications,” Chemical Society Reviews 49 (2020): 5850–5884, 10.1039/d0cs00318b. 32662499

[12] A. Farokh Payam and A. Passian , “Imaging beyond the Surface Region: Probing Hidden Materials via Atomic Force Microscopy,” Science Advances 9, no. 26 (2023): eadg8292. 37379392 10.1126/sciadv.adg8292 PMC10306303

[13] R. Garcia and E. T. Herruzo , “The Emergence of Multifrequency Force Microscopy,” Nature Nanotechnology 7 (2012): 217–226, 10.1038/nnano.2012.38. 22466857

[14] V. Pukhova and G. Ferrini , “Multi‐Frequency Data Analysis in AFM by Wavelet Transform,” in IOP Conference Series: Materials Science and Engineering (Institute of Physics Publishing, 2017), 256.

[15] Z. Wang , J. Qian , Y. Li , et al., “Wavelet Analysis of Higher Harmonics in Tapping Mode Atomic Force Microscopy,” Micron 118 (2019): 58–64. 30597428 10.1016/j.micron.2018.12.007

[16] J. Carracedo‐Cosme and R. Pérez , “Molecular Identification with Atomic Force Microscopy and Conditional Generative Adversarial Networks,” NPJ Computational Materials 10, no. 1 (2024): 19.

[17] A. Belianinov , S. V. Kalinin , and S. Jesse , “Complete Information Acquisition in Dynamic Force Microscopy,” Nature Communications 6 (2015): 6550. 10.1038/ncomms7550 25766370

[18] M. G. Ruppert and S. O. R. Moheimani , “High‐Bandwidth Multimode Self‐Sensing in Bimodal Atomic Force Microscopy,” Beilstein Journal of Nanotechnology 7 (2016): 284–295. 26977385 10.3762/bjnano.7.26 PMC4778537

[19] M. G. Ruppert , D. M. Harcombe , M. R. P. Ragazzon , S. O. Reza Moheimani , and A. J. Fleming , “A Review of Demodulation Techniques for Amplitude‐Modulation Atomic Force Microscopy,” Beilstein Journal of Nanotechnology 8 (2017): 1407–1426, 10.3762/bjnano.8.142. 28900596 PMC5530615

[20] M. Ayat , M. A. Karami , S. Mirzakuchaki , and A. Beheshti‐Shirazi , “Design of Multiple Modulated Frequency Lock‐In Amplifier for Tapping‐Mode Atomic Force Microscopy Systems,” IEEE Transactions on Instrumentation and Measurement 65 (2016): 2284–2292.

[21] K. S. Karvinen and S. O. R. Moheimani , “A High‐Bandwidth Amplitude Estimation Technique for Dynamic Mode Atomic Force Microscopy,” Review of Scientific Instruments 85 (2014), 10.1063/1.4865841. 24593371

[22] T. Ando , T. Uchihashi , and T. Fukuma , “High‐Speed Atomic Force Microscopy for Nano‐Visualization of Dynamic Biomolecular Processes,” Progress in Surface Science 83 (2008): 337–437, 10.1016/j.progsurf.2008.09.001.

[23] M. Checa , A. S. Fuhr , C. Sun , et al., “High‐Speed Mapping of Surface Charge Dynamics Using Sparse Scanning Kelvin Probe Force Microscopy,” Nature Communications 14 (2023): 7196. 10.1038/s41467-023-42583-x PMC10632481 37938577

[24] M. Carmichael , R. Vidu , A. Maksumov , A. Palazoglu , and P. Stroeve , “Using Wavelets to Analyze AFM Images of Thin Films: Surface Micelles and Supported Lipid Bilayers,” Langmuir 20 (2004): 11557–11568. 15595784 10.1021/la048753c

[25] G. Malegori and G. Ferrini , “Wavelet Transforms to Probe Long‐and Short‐Range Forces by Thermally Excited Dynamic Force Spectroscopy,” Nanotechnology 22 (2011): 195702. 21430315 10.1088/0957-4484/22/19/195702

[26] F. Banfi and G. Ferrini , “Wavelet Cross‐Correlation and Phase Analysis of a Free Cantilever Subjected to Band Excitation,” Beilstein Journal of Nanotechnology 3 (2012): 294–300. 22497003 10.3762/bjnano.3.33 PMC3323919

[27] E. A. López‐Guerra , F. Banfi , S. D. Solares , and G. Ferrini , “Theory of Single‐Impact Atomic Force Spectroscopy in Liquids with Material Contrast,” Scientific Reports 8 (2018): 7534. 29760518 10.1038/s41598-018-25828-4 PMC5951954

[28] V. Pukhova , F. Banfi , and G. Ferrini , “Complex Force Dynamics in Atomic Force Microscopy Resolved by Wavelet Transforms,” Nanotechnology 24 (2013): 505716. 24285087 10.1088/0957-4484/24/50/505716

[29] V. Pukhova , F. Banfi , and G. Ferrini , “Transient Eigenmodes Analysis of Single‐Impact Cantilever Dynamics Combining Fourier and Wavelet Transforms,” Nanotechnology 26 (2015): 1–10. 10.1088/0957-4484/26/17/175701 25837684

[30] V. Pukhova , F. Banfi , and G. Ferrini , “Energy Dissipation in Multifrequency Atomic Force Microscopy,” Beilstein Journal of Nanotechnology 5 (2014): 494–500. 24778976 10.3762/bjnano.5.57 PMC3999740

[31] Z. Wang , J. Qian , Y. Li , et al., “Time‐Frequency Analysis of the Tip Motion in Liquids Using the Wavelet Transform in Dynamic Atomic Force Microscopy,” Nanotechnology 29 (2018): 385702. 29957597 10.1088/1361-6528/aad031

[32] A. Farokh Payam , P. Biglarbeigi , A. Morelli , P. Lemoine , J. McLaughlin , and D. Finlay , “Data Acquisition and Imaging Using Wavelet Transform: A New Path for High Speed Transient Force Microscopy,” Nanoscale Advances 3 (2021): 383–398. 36131753 10.1039/d0na00531b PMC9417248

[33] P. Biglarbeigi , A. Morelli , S. Pauly , et al., “Unraveling Spatiotemporal Transient Dynamics at the Nanoscale via Wavelet Transform‐Based Kelvin Probe Force Microscopy,” ACS Nano 17 (2023): 21506–21517. 37877266 10.1021/acsnano.3c06488 PMC10655243

[34] P. Biglarbeigi , A. Morelli , G. Bhattacharya , et al., “Incongruous Harmonics of Vibrating Solid‐Solid Interface,” Small 21 (2025): 2409410, 10.1002/smll.202409410. 39552010 PMC11899492

[35] P. Biglarbeigi , G. Bhattacharya , D. Finlay , and A. F. Payam , “Nonlinear Harmonics: A Gateway to Enhanced Image Contrast and Material Discrimination,” Advanced Science 12 (2025); 2411556. 39876697 10.1002/advs.202411556 PMC11923995

[36] L. P. A. Arts and E. L. van den Broek , “The Fast Continuous Wavelet Transformation (fCWT) for Real‐Time, High‐Quality, Noise‐Resistant Time–frequency Analysis,” Nature Computational Science 2 (2022): 47–58. 38177705 10.1038/s43588-021-00183-z PMC10766549

[37] A. T. Walden and A. C. Cristan , “The Phase–corrected Undecimated Discrete Wavelet Packet Transform and Its Application to Interpreting the Timing of Events,” Proceedings of the Royal Society of London. Series A: Mathematical, Physical and Engineering Sciences 454 (1998): 2243–2266.

[38] Z. Tu , S. Choudhury , M. J. Zachman , et al., “Fast Ion Transport at Solid–solid Interfaces in Hybrid Battery Anodes,” Nature Energy 3 (2018): 310–316.

[39] Y. R. Shen , “Surface Properties Probed by Second‐Harmonic and Sum‐Frequency Generation,” Nature 337 (1989): 519–525.

[40] M. Österberg , K. A. Henn , M. Farooq , and J. J. Valle‐Delgado , “Biobased Nanomaterials—the Role of Interfacial Interactions for Advanced Materials,” Chemical Reviews 123 (2023): 2200–2241. 36720130 10.1021/acs.chemrev.2c00492 PMC9999428

[41] F. Variola , “Atomic Force Microscopy in Biomaterials Surface Science,” Physical Chemistry Chemical Physics 17 (2015): 2950–2959. 25523021 10.1039/c4cp04427d

[42] D. Forchheimer , R. Forchheimer , and D. B. Haviland , “Improving Image Contrast and Material Discrimination with Nonlinear Response in Bimodal Atomic Force Microscopy,” Nature Communications 6 (2015): 6270. 10.1038/ncomms7270 PMC4346977 25665933

[43] A. Raman , J. Melcher , and R. Tung , “Cantilever Dynamics in Atomic Force Microscopy,” Nano Today 3 (2008): 20–27.

[44] R. W. B. Stark , “Higher Harmonics, and Chaos in AFM,” Materials Today 13 (2010): 24–32.

[45] M. Ziatdinov , O. Dyck , A. Maksov , et al., “Deep Learning of Atomically Resolved Scanning Transmission Electron Microscopy Images: Chemical Identification and Tracking Local Transformations,” ACS Nano 11 (2017): 12742–12752. 29215876 10.1021/acsnano.7b07504

[46] R. Ma , E. D. Sun , and J. Zou , “A Spectral Method for Assessing and Combining Multiple Data Visualizations,” Nature Communications 14 (2023): 780. 10.1038/s41467-023-36492-2 PMC9922271 36774377

[47] L. Collins , M. Ahmadi , T. Wu , B. Hu , S. V. Kalinin , and S. Jesse , “Breaking the Time Barrier in Kelvin Probe Force Microscopy: Fast Free Force Reconstruction Using the G‐Mode Platform,” ACS Nano 11 (2017): 8717–8729. 28780850 10.1021/acsnano.7b02114

[48] Z. Schumacher , A. Spielhofer , Y. Miyahara , and P. Grutter , “The Limit of Time Resolution in Frequency Modulation Atomic Force Microscopy by a Pump‐Probe Approach,” Applied Physics Letters 110 (2017): 053111.

[49] J. Murawski , T. Mönch , P. Milde , et al., “Tracking Speed Bumps in Organic Field‐Effect Transistors via Pump‐Probe Kelvin‐Probe Force Microscopy,” Journal of Applied Physics 118, 2015.

