# Supplementary material for: Wavelet Transform‐Based Atomic Force Microscopy: A Computational Paradigm for Dynamic Nanoscale Imaging and Characterisation
Source: Small Sci. 2026 Jul 26;6(8):e70350. doi: 10.1002/smsc.70350 (PMC13410550; doi:10.1002/smsc.70350)
Supplement: Supplementary file 1 — Supplementary Material [file SMSC-6-e70350-s001.pdf]

Supplementary Information

Wavelet Transform–Based Atomic Force Microscopy: A Computational Paradigm for Dynamic Nanoscale Imaging and Characterization

Pardis Biglarbeigi[1,2,*], Navneet Soin[3,4], Amit Kumar[5], Dewar Finlay[6] and Amir Farokh Payam[6,*]

[1]Department of Pharmacology & Therapeutics, University of Liverpool, Whelan Building, Liverpool, England, L69 3GE, United Kingdom
[2]PulseAI, Belfast BT1 6PJ, United Kingdom
[3]School of Science, Computing and Emerging Technologies (SoSCET), Swinburne University of Technology, Hawthorn, VIC 3122, Australia
[4]School of Science, RMIT University, Melbourne, VIC 3000, Australia
[5]Centre for Quantum Materials and Technologies (CQMT), School of Mathematics and Physics, Queen's University Belfast, Belfast BT7 1NN, United Kingdom
[6]School of Engineering, Ulster University, York Street, Belfast, Northern Ireland, BT15 1AP, United Kingdom

Emails: P.Biglarbeigi@liverpool.ac.uk, a.farokh-payam@ulster.ac.uk

1. Cross Wavelet Transform (XWT)

XWT quantifies local interactions between two signals, such as cantilever response and drive, with the local phase lag calculated as:

$$W_{xy}(t,s) = W_x(t,s)\, W_y^*(t,s) \tag{1}$$

where W_x and W_y represent the wavelet transforms of the cantilever response and drive, respectively. While, the wavelet coherence,

$$R^2(t,s) = \frac{|S(s^{-1}W_{xy}(t,s))|^2}{S(s^{-1}|W_x(t,s)|^2)\, S(s^{-1}|W_y(t,s)|^2)}, \tag{2}$$

measures the localised correlation between the drive signal and response after smoothing by $S(\cdot)$. Wavelet coherence evaluates the time-frequency correlation between signals, providing a measure of localised coherence between 0 and 1. For coherence value greater than 0.5, the phase difference between the two signals, ϕ_{xy}, can be calculated:

$$\phi_{xy}(t,s) = \arctan\frac{\Im\{W_{xy}(t,s)\}}{\Re\{W_{xy}(t,s)\}} \tag{3}$$

2. Singular value decomposition (SVD)

SVD, which underpins Principal Component Analysis (PCA), is used to remove uncorrelated noise. This approach is particularly effective in experiments involving repeated transient patterns, such as pulsed excitations, where consistent features could be retained while uncorrelated noise was suppressed. As such, the deflection signal is initially reshaped into a two-dimensional $n \times p$ matrix A, with each column corresponding to an individual pulse. SVD is then applied as:

$$A = USV^T \tag{4}$$

where U is an $n \times n$ unitary matrix containing the left singular vectors, S is an $n \times p$ diagonal matrix, whose non-zero entries represent the singular values, and V^T is the transpose of a $p \times p$ unitary matrix containing the right singular vectors. The dominant principal components, corresponding to the largest singular values in S, were selected to reconstruct the denoised signal, while components associated with smaller singular values were discarded as noise, following the scree-plot "elbow" criterion.

3. Objective image quality metrics

Objective image quality metrics quantitatively assess the relationship between interaction forces and the material properties of each sample. These metrics provide numerical evaluations of image quality based on statistical parameters and, where applicable, reference images. Three widely used objective metrics are utilised:

Peak Signal-to-Noise Ratio (PSNR): quantifies image quality by comparing the maximum possible pixel intensity to the noise level, expressed as the mean squared error (MSE) between the processed image and a reference image. Higher PSNR values indicate improved image quality and contrast, whereas lower values correspond to greater numerical differences between the two images.

$$PSNR = 10\log\left(\frac{A_{max}^2}{MSE}\right) \tag{5}$$

where A_{max} denotes the maximum possible value of the image.

Structural Similarity Index Measure (SSIM): evaluates image similarity based on perceived structural information rather than simple pixel-wise differences. It considers three components of luminance, contrast, and structural correlation, to capture changes in image content that align with the human visual system. For this analysis, all weighting parameters for these components are set to unity, following standard practice. SSIM is defined as:

$$SSIM(A, \hat{A}) = [l(A, \hat{A})]^{\alpha}\ [c(A, \hat{A})]^{\beta}\ [s(A, \hat{A})]^{\gamma} \tag{6}$$

where l, c and s denote the luminance, contrast, and the structural terms of the images. $\alpha > 0$, $\beta > 0$, and $\gamma > 0$ are constant values that are adjusted based on relative importance of each component, and for simplicity they are considered to be 1.

Correlation Coefficient: quantifies the degree of linear association between the processed and reference images. A correlation value closer to one indicates a stronger similarity in image features, while lower values reflect increased dissimilarity. The correlation coefficient between two images is calculated as:

$$r = \frac{\sum_{i=1}^{X}\sum_{j=1}^{Y}(A(i,j)-mean(A))(\hat{A}(i,j)-mean(\hat{A}))}{\sum_{i=1}^{X}\sum_{j=1}^{Y}(A(i,j)-mean(A))^2\ \sum_{i=1}^{X}\sum_{j=1}^{Y}(\hat{A}(i,j)-mean(\hat{A}))^2} \tag{7}$$

4. Image Contrast Measures

From the reconstructed harmonic images, quantitative evaluation is carried out using complementary contrast measures: the sample contrast index (c), image standard deviation (SD), entropy (H), and spatial frequency (f_x, f_y).

Contrast index, (c), is derived from normalised histograms of the amplitude images, denoted by $\mathcal{N}(.)$, modelled using Gaussian mixture distributions.

$$p(x) = p\,\mathcal{N}(\mu_1, \sigma_1^2) + (1-p)\,\mathcal{N}(\mu_2, \sigma_2^2), \tag{8}$$

with p and $1-p$ denoting the pixel fractions of the two phases. The contrast is then quantified as,

$$c = \frac{|\mu_1 - \mu_2|}{\sigma_1 + \sigma_2}, \tag{9}$$

where larger values of c correspond to greater separability between constituent phases. For more complex heterogeneous systems, mixtures of more than two Gaussians are considered to better capture multimodal contrast behaviour.

Standard deviation (SD) measures global intensity variation and is defined as:

$$SD = \frac{1}{XY}\sqrt{\sum_{i=1}^{X} \sum_{j=1}^{Y} (F(i,j) - \mu)^2}, \tag{10}$$

where *X* and *Y* represent the dimensions, *F*(*i*, *j*) represents the pixel value located in (*i*, *j*) and μ is the mean pixel value of the image,

Entropy (H), quantifies image information content and texture complexity and is defined as:

$$H = -\sum_i p(a_i) \log p(a_i), \tag{11}$$

where $p(a_i)$ shows the histogram of the intensity levels in the image.

Spatial frequency analysis, further, provides sensitivity to localised periodic variations in the intensity maps, distinguishing fine structural features from broader topographic changes.

5. Computational Protocol: WT_AFM_harmonic_extraction

The function supporting this study for calculation of harmonics simultaneously is available at *https://github.com/pardis-pb/WT-AFM.git*.

5.1. Scope:

This protocol describes the computational procedure implemented in the MATLAB function `WT_AFM_harmonic_extraction`, which forms a core component of the wavelet-based signal processing pipeline described in the accompanying manuscript. The function extracts the instantaneous amplitude envelopes of user-specified harmonic frequency components from a raw AFM cantilever deflection signal, and additionally recovers the instantaneous phase of the fundamental drive frequency relative to a reference drive signal. These

quantities are central to multi-frequency AFM image contrast and nanomechanical property mapping.

The method combines three wavelet-domain operations: (i) a Maximal Overlap Discrete Wavelet Packet Transform (MODWPT) for frequency-selective band isolation, (ii) a Continuous Wavelet Transform (CWT) for instantaneous amplitude recovery within each isolated band, and (iii) Wavelet Coherence (WCO) for phase estimation relative to the reference drive signal.

5.2. Software requirements:

The following software environment is required to execute this protocol:

Component	Requirement
MATLAB	R2021b or later (arguments block syntax required)
Wavelet Toolbox	Required for `modwptdetails`, `cwt`, and `wcoherence` functions
Operating System	Windows, macOS, or Linux (any platform supporting MATLAB)
Memory	Minimum 8 GB RAM recommended; 16 GB or more for signals exceeding 10·ChunkSize samples
Toolbox licence	The function checks licence availability at runtime; an error is raised if the Wavelet Toolbox is not licenced

5.3. Input Parameters

Required Inputs

Parameter	Type / Size	Description	Default
`sig`	1×N or N×1 double	Cantilever deflection signal under analysis. Must be real, finite, and non-empty.	—
`fd`	1×N or N×1 double	Reference drive signal. Must have the same number of samples as sig. Used solely for wavelet coherence phase estimation.	—
`fs`	positive scalar (Hz)	Sampling frequency in Hz.	—
`f1`	positive scalar (Hz)	Fundamental excitation frequency. Harmonics are computed at k·f1 for k = 1, 2, ..., num_harmonics.	—
`num_harmonics`	positive integer	Number of harmonics of f1 to extract. Harmonics exceeding the Nyquist frequency are automatically excluded with a warning.	—
`f2`	positive scalar (Hz)	Second (independent) frequency of interest, e.g. a higher eigenmode resonance. Extracted as an additional row in amp_all.	—

Optional Name-Value Inputs

Parameter	Type / Size	Description	Default
Wavelet	char	Wavelet family string accepted by modwptdetails. Higher-order Daubechies wavelets provide sharper frequency localisation.	'db45'
WPTLevel	positive integer	Decomposition depth of the MODWPT. Increasing the level improves frequency resolution at the cost of computational time and memory.	7
Padding	non-negative int	Number of zero-padding samples appended at both ends of each processing chunk to suppress boundary artefacts.	2000
ChunkSize	positive integer	Number of signal samples processed per loop iteration. Reduce to avoid out-of-memory errors for very long recordings.	$1×10^6$
BandwidthExp	positive scalar	Controls the CWT frequency search window around each target: window half-width = f_target×(exp(B)−1)/2. Smaller values give tighter windows.	0.1
Verbose	logical	If true, progress information is printed to the command window during chunked processing.	true

Output Variables

Output	Dimensions	Description
amp_all	(num_harmonics+1) × N	Instantaneous amplitude matrix. Row k (k = 1…num_harmonics) contains the envelope at k·f1; the final row contains the envelope at f2. Units are the same as the input signal.
phase_f1	1 × N	Instantaneous phase (radians) of the wavelet coherence between sig and fd evaluated at f1. Values lie in the interval (−π, π].
freq_labels	1 × (num_harmonics+1) cell	Cell array of descriptive strings labelling each row of amp_all, e.g. {'1f1 (29566 Hz)', '2f1 (59132 Hz)', ..., 'f2 (219345 Hz)'}.

5.4. Algorithmic Procedure

The processing pipeline comprises six sequential stages, illustrated conceptually below.

Stage 1: Input Validation and Pre-processing

1. Row-vector orientation is enforced for both sig and fd.
2. Signal and drive lengths are verified to be equal; a fatal error is raised on mismatch.
3. f1 and f2 are checked against the Nyquist frequency (fs/2); an error is raised if either equals or exceeds this limit.

4. Harmonics of f1 that exceed the Nyquist frequency are silently pruned and a warning is issued.

5. Proximity warnings are issued when f1 and f2 are within 1% of each other, or when any harmonic of f1 is within 1% of f2, as frequency-band overlap may degrade accuracy in these regimes.

6. Availability of the MATLAB Wavelet Toolbox licence is verified before any computation is performed.

Stage 2: WPT Sub-band Identification

A probe segment (at most 10^6 samples, drawn from the centre of the recording) is submitted to `modwptdetails` at the specified wavelet and decomposition level. The returned centre frequencies and inferred bandwidths are used to assign each target frequency (harmonics of f1 and f2) to the most appropriate WPT sub-band. If a target does not fall within any defined sub-band, the nearest sub-band by centre frequency is selected and a warning is issued.

Stage 3: Chunked Processing Loop

The full signal is divided into non-overlapping chunks of `ChunkSize` samples to bound peak memory usage. Each chunk is processed independently; zero-padding of Padding samples is applied at both boundaries before decomposition and stripped after, ensuring that boundary artefacts do not contaminate the retained samples.

Stage 4: Wavelet Packet Decomposition

For each padded chunk, `modwptdetails` is applied to obtain the MODWPT coefficient matrix. Only the rows corresponding to the previously identified sub-band indices are retained (one row per target frequency), yielding a compact matrix of size `(num_harmonics+1)×(chunk_length+2×Padding)`.

Stage 5: CWT Amplitude Extraction

Each retained sub-band signal is submitted individually to the CWT. The frequency search window is centred on the target frequency with half-width proportional to `f_target×(exp(BandwidthExp)−1)/2`. The scale closest to the target frequency is identified by minimum absolute difference, and the modulus of the corresponding CWT coefficient row is taken as the instantaneous amplitude envelope. Post-padding samples are removed and the amplitude values are written into the appropriate slice of `amp_all`.

Stage 6: Wavelet Coherence Phase Estimation

Wavelet coherence between the padded chunk of sig and the corresponding padded chunk of fd is computed via `wcoherence`. The complex coherence at the scale nearest to f1 is extracted, and its argument (angle) provides the instantaneous phase of the drive-to-response relationship. Post-padding samples are removed and written into `phase_f1`.

5.5. Minimal Working Example

The following self-contained MATLAB script reproduces a typical call:

```
% --- Parameters ---
fs= 1e6;    % sampling frequency (Hz)
f1= 29.566e3;    % first eigenmode resonance (Hz)
```

```
f2= 219.345e3;   % second eigenmode resonance (Hz)
t= (0: fs-1) / fs;    % time vector (1 s)

% --- Synthetic test signals ---
sig=sin(2*pi*f1*t)+ 0.3*sin(2*pi*f2*t) + 0.05*randn(size(t));
fd=sin(2*pi*f1*t + 0.4) % drive signal with known phaseoffset

% --- Run extraction ---
[amp, phi, labels] = WT_AFM_harmonic_extraction(sig, fd, fs,
f1, 3, f2);

% --- Plot ---
nRow = size(amp, 1) + 1;
figure;
for k = 1 : size(amp, 1)
    subplot(nRow, 1, k);
    plot(t, amp(k,:));  ylabel(labels{k});  grid on;
end
subplot(nRow, 1, nRow);
plot(t, phi);  ylabel('Phase f1 (rad)');  xlabel('Time (s)');
grid on;
```

5.7. Warnings, Edge Cases, and Known Limitations

Run-time Warnings

Warning ID	Cause and Recommended Action
`WT_AFM_harmonic_extraction:harmonicsAboveNyquist`	One or more requested harmonics exceed fs/2. The function automatically reduces `num_harmonics`. Verify fs and f1 are correct.
`WT_AFM_harmonic_extraction:f1f2TooClose`	f1 and f2 are within 1% of each other. Their WPT bands may overlap; results may be unreliable. Choose spectrally distinct frequencies if possible.
`WT_AFM_harmonic_extraction:harmonicNearF2`	A harmonic of f1 lies within 1% of f2. The affected row of `amp_all` may contain leakage from both components.
`WT_AFM_harmonic_extraction:shortSignal`	Signal is shorter than one processing chunk. Boundary effects may be significant. Consider increasing Padding or reducing `ChunkSize`.
`WT_AFM_harmonic_extraction:bandNotFound`	A target frequency does not coincide with any WPT sub-band. The nearest sub-band is used; increase `WPTLevel` to improve frequency resolution.
`WT_AFM_harmonic_extraction:f2BandNotFound`	f2 does not coincide with any WPT sub-band. See :`bandNotFound` above.

Known Limitations

- The function processes all chunks sequentially and does not currently exploit MATLAB parallel computing infrastructure (e.g. parfor or GPU arrays). Parallelisation across chunks is left to the user.

- Memory consumption scales with `ChunkSize + 2×Padding`. For recordings longer than approximately 10^8 samples, it is advisable to reduce `ChunkSize` to $5×10^5$ or lower.

- The phase output (phase_f1) is derived from the argument of the complex wavelet coherence and may exhibit phase wrapping artefacts at transitions between chunks if the coherence magnitude is low. Unwrapping (e.g. using MATLAB's unwrap) is recommended for continuous-phase applications.

- Frequency resolution of the WPT is determined jointly by `WPTLevel`, the selected wavelet, and fs. At the default level of 7, the number of sub-bands is 2^7 = 128; finer resolution requires a higher level or a longer wavelet filter (higher wavelet order), both of which increase computation time.

- The probe segment used in Stage 2 is drawn from the centre of the recording. For non-stationary signals in which spectral content changes significantly over time, the assigned sub-band indices may be suboptimal for portions of the signal far from the probe region.

5.8 Parameter Selection Guidance

Wavelet and `WPTLevel`

The Daubechies db45 wavelet used as the default provides excellent frequency localisation owing to its high number of vanishing moments and its correspondingly long filter length. Lower-order wavelets (e.g. db4, db8) reduce computation time but may introduce greater spectral leakage between adjacent sub-bands. Increasing `WPTLevel` from 7 to 8 doubles the number of frequency sub-bands, halving the bandwidth of each, and is recommended when harmonics of f1 are closely spaced relative to fs.

`Padding`

The default value of 2000 samples is sufficient for the db45 wavelet at level 7 under AFM sampling conditions of f_s = 1 MHz. For lower sampling rates, shorter signals, or higher wavelet orders, the filter length increases and a larger padding value may be required.

`BandwidthExp`

The default value of 0.1 corresponds to a CWT search window of approximately ±5% around each target frequency. This setting is appropriate when harmonics are well-separated. Reducing `BandwidthExp` (e.g. to 0.05) narrows the window and may improve specificity when harmonics are closely spaced; however, very narrow windows risk missing the target scale when the resonance frequency is shifted by tip-sample interactions.

6. Wavelet selection framework

Wavelet family	Signal structure best represented	Mathematical strength	Time-Frequency characteristics	Physical information optimally extracted	Preferred AFM/KPFM application(s)
Analytic Morse	Non-stationary harmonics; time-varying amplitude and phase modulated oscillations	Fully analytic wavelet; independently tuneable symmetry and time-bandwidth product	Adjustable localization via γ and P^2; enables simultaneous tracking of multiple harmonic branches	Time-dependent CPD, electrostatic force harmonics (ω, 2ω, 3ω), transient charge trapping/detrapping, nanoscale electrostatic dynamics	Open-Loop KPFM, Dynamic Harmonic AFM, Transient Electrostatic Mapping
Analytic Morlet	Narrowband resonance signals with smooth frequency modulation	Near-optimal localisation; Gaussian-modulated complex sinusoid	Excellent frequency localisation with moderate temporal resolution	Resonance frequency shifts (Δf), force gradients, local dissipation mechanisms	FM-AFM, Dissipation Spectroscopy, Resonance Tracking
Daubechies	Sparse transient events, impulse response	Compact support, orthogonality, high vanishing moments, energy-preserving decomposition	Multiresolution discrete decomposition; strong temporal localisation	adhesion rupture, intermittent-contact transition, force pulses embedded in broadband noise	High-Speed Topography, Transient Force Microscopy, DWT Denoising
Symlets	Similar to Daubechies but with improved phase symmetry	Near-linear phase response	Balanced temporal and spectral localisation	Quantitative transient reconstruction with reduced phase bias	Transient Force Imaging, Feature Extraction, Harmonic Image Fusion
Haar	Step discontinuity and abrupt transitions	Shortest compact support	Maximum temporal localisation, poor spectral selectivity	Jump-to-contact events, contact-loss transitions, nanoscale friction discontinuities	Pulsed Force Mode, Contact Transition Detection, Stick-Slip Dynamics

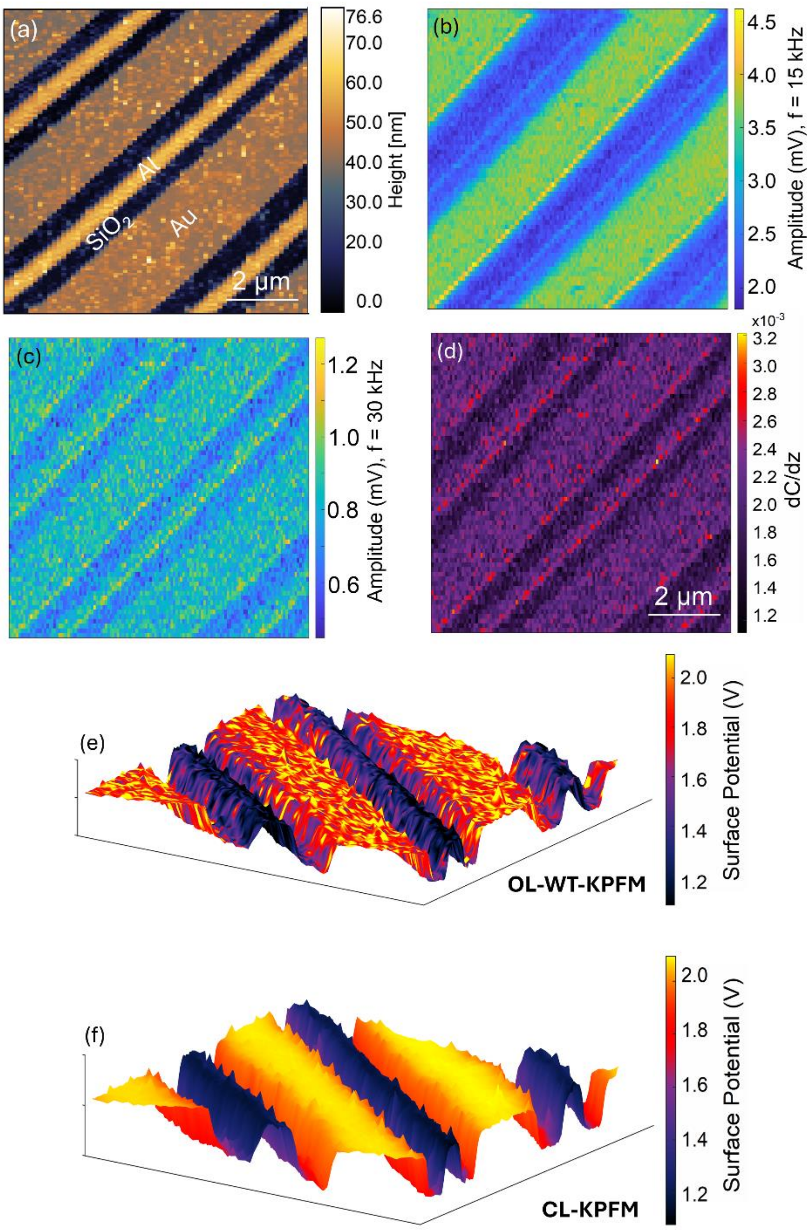

Fig. S1: *(a) Topography of the KPFM-EFM calibration sample consisting of alternate Au/Al tracks on SiO_2 substrate. For this sample DC-biased at 2 V, the OL-WT-KPFM technique extracts the (b) first harmonic (A_{ω}), at the cantilever excitation frequency and (c) second harmonic ($A_{2\omega}$) from the collected data stream. (d) Map of the localised capacitance gradient ($\partial C/\partial z$) across the surface and (e) the 3D overlay mapping of the absolute surface potential onto the topography calculated offline via the OL-WT-KPFM framework. (f) Corresponding 3D surface potential map acquired using the standard LIA-based CL-KPFM for cross-validation.*
